# Many genes in fish have species-specific asymmetric rates of molecular evolution

**DOI:** 10.1186/1471-2164-7-20

**Published:** 2006-02-08

**Authors:** Dirk Steinke, Walter Salzburger, Ingo Braasch, Axel Meyer

**Affiliations:** 1 Lehrstuhl für Zoologie und Evolutionsbiologie, Department of Biology, University of Konstanz, D-78457 Konstanz, Germany; 2 Center of Junior Research Fellows, University of Konstanz, D-78457 Konstanz, Germany; 3 Department of Physiological Chemistry I, Biozentrum, University of Wuerzburg, Germany

## Abstract

**Background:**

Gene and genome duplication events increase the amount of genetic material that might then contribute to an increase in the genomic and phenotypic complexity of organisms during evolution. Thus, it has been argued that there is a relationship between gene copy number and morphological complexity and/or species diversity. This hypothesis implies that duplicated genes have subdivided or evolved novel functions compared to their pre-duplication proto-orthologs. Such a functional divergence might be caused by an increase in evolutionary rates in one ortholog, by changes in expression, regulatory evolution, insertion of repetitive elements, or due to positive Darwinian selection in one copy. We studied a set of 2466 genes that were present in *Danio rerio*, *Takifugu rubripes*, *Tetraodon nigroviridis *and *Oryzias latipes *to test (i) for forces of positive Darwinian selection; (ii) how frequently duplicated genes are retained, and (iii) whether novel gene functions might have evolved.

**Results:**

25% (610) of all investigated genes show significantly smaller or higher genetic distances in the genomes of particular fish species compared to their human ortholog than their orthologs in other fish according to relative rate tests. We identified 49 new paralogous pairs of duplicated genes in fish, in which one of the paralogs is under positive Darwinian selection and shows a significantly higher rate of molecular evolution in one of the four fish species, whereas the other copy apparently did not undergo adaptive changes since it retained the original rate of evolution. Among the genes under positive Darwinian selection, we found a surprisingly high number of ATP binding proteins and transcription factors.

**Conclusion:**

The significant rate difference suggests that the function of these rate-changed genes might be essential for the respective fish species. We demonstrate that the measurement of positive selection is a powerful tool to identify divergence rates of duplicated genes and that this method has the capacity to identify potentially interesting candidates for adaptive gene evolution.

## Background

Biology is a discipline rooted in comparisons. Comparative studies have led to the assembly of a detailed catalogue of biological similarities and, also, of differences between species, yielding insights into the mechanisms by which organisms and their genomes adapt to a wide range of ecological niches. Genomics is the most recent biological discipline to employ comparison-based approaches. During the last five years, whole genome sequences have become available for several vertebrates: e.g., human, mouse, rat, chicken, zebrafish, and two pufferfish species (*Takifugu rubripes *and *Tetraodon nigroviridis*) [1-6]. The increasing wealth of sequence data allows whole genome comparisons for the study of the evolutionary forces that shape genomes [7]. Comparative strategies have identified chromosomal blocks of DNA sequences that are conserved over long evolutionary time spans. Such a degree of evolutionary conservation has, for example, been a powerful guide in sorting functional from non-functional DNA [8-11] and to assign putative gene function.

Ray-finned fishes, which comprise ~25,000 extant species [12], are the most species-rich group of vertebrates. They show enormous differences in their morphology and adaptations to divergent environments. Their sister group are the lobe-finned fishes, which include the other half of all bony vertebrates, such as coelacanths, lungfishes and the tetrapods (amphibian, reptiles, birds and mammals). The ray-finned fishes and the lobe-finned fishes diverged between 400–450 million years ago [13]. Although this large evolutionary distance would imply that only a rather small fraction of the functional portions of their genomes are shared, comparative studies revealed that most human coding sequences (~91%) are homologous to genes in fish [5]. Natural selection is known to leave its footprint on protein-coding sequences in a genome by affecting rates of silent and replacement rates differentially. In sequences that have evolved under positive selection, the number of retained mutations is closer to those that arose by mutation than under purifying selection where amino acid replacement mutations are selected against.

It has been suggested that the large number of fish species and their tremendous morphological diversity might be causally related to a genome duplication event that is specific to the teleost lineage [14-21]. Since gene and genome duplication events are likely to increase the genetic raw-material, it has been speculated that there is a relationship between gene copy number and morphological complexity and, by extension, also species diversity [22,23]. This would imply that one copy of a duplicated gene has diverged from the roles of the pre-duplication ortholog. Such a divergence could be demonstrated by an increase in evolutionary rate, expression differences, regulatory evolution, and/or by evidence for positive Darwinian selection. Duplicated genes may be redundant after the duplication event, which means that inactivation of one of the two duplicates might have little or no effect on the phenotype [24-26]. Therefore, since at least one of the copies is free from any functional constraint, mutations in this gene-copy might be selectively neutral, having the potential to turn one copy into a non-functional pseudogene. Alternatively, one of the duplicates might adopt a new function through neofunctionalization [22,27,28], or the ancestral function might get divided between the paralogs (subfunctionalization) [29,30]. Recent studies revealed that subfunctionalization can occur rapidly and is often accompanied by prolonged and substantial rates of neofunctionalization in a large proportion of duplicated genes. Thus, a new model, termed sub-neofunctionalization (SNF) has been proposed [31,32]. Other authors argue, that the evolution of new functions may start with the duplication of an existing gene in a sense a preadaptation for that function, followed by a period of evolution among the gene copies, resulting in the preservation of the most effective variant and the 'pseudogenization' and eventual loss of the remaining copies [33].

Post-duplication secondary gene loss is relatively frequent. However, it has been estimated that, nonetheless, ~20%–50% of paralogous genes are retained for longer evolutionary time spans after a genome duplication event [34,35]. A selective advantage due to a new and possibly unique function seems to be sufficient to retain a copy and to prevent degenerative substitutions that would ultimately drive the other copy to become a pseudogene. Among other factors positive Darwinian selection can be responsible for functional divergence between two duplicates (e.g., [36-38]). When a gene with multiple functions is duplicated, the duplicates are redundant only for as long as each copy retains the ability to perform all ancestral roles [29,30]. According to the duplication-degeneration-complementation (DCC) model [30], degenerative mutations preserve rather than disrupt duplicated genes, but also change their functions or at least restrict their original functions which later might become more specialized.

In the current study, simultaneous sequence comparisons of the entire protein-coding portions of the genomes of four fish model species (*Danio rerio*, *Takifugu rubripes*, *Tetraodon nigroviridis *and *Oryzias latipes*) with the human outgroup genome were conducted in order to study the evolutionary extent of sequence conservation and divergence in duplicated fish genes. To facilitate gene identification for functional genomic studies, each data set has been annotated using the structured vocabulary provided by the Gene Ontology Consortium (2001), based on molecular studies of the gene function in *Homo sapiens*. To detect lineage-specific evolutionary processes, we attempted to identify genes that seem to have evolved with significantly divergent (slower or faster) rates of amino acid substitution in one particular species. To this end, we applied a non-parametric relative rate test [39]. Duplicated genes identified with this approach were further studied to test for evidence of positive Darwinian selection and whether the hypothesis for the retention of duplicated genes and the evolution of novel gene functions in one copy is supported. To test whether sequences have been subjected to positive Darwinian selection, the ratio of the proportion of radical nonsynonymous difference (dR) per radical nonsynonymous site and the proportion of conservative nonsynonymous site (dC) was calculated using the an approach of Hughes *et al*. [40]. Although different methods have been developed to detect positive selection based on the rate of nonsynonymous (dN) and synonymous substitutions (dS), we note that this ratio can be possibly used to detect positive selection for recently diverged genes only (30–50 MYA) as demonstrated in previous studies [41,42]. It has also been argued that positive selection is of an episodic nature. Meaning that, after a period of positive selection, purifying selection usually blurs the substitution pattern that is indicative of positive selection [38,43]. Since the method by Hughes *et al. *[40] compares nonsynonymous sites and the resulting amino acid changes only, positive selection would need to be active for a much longer period. It should be noted though, that this method may be less sensitive than methods based on the dN/dS ratio [43,44]. Furthermore, a recent study revealed that the dR/dC measure is influenced by the transition/transversion ratio and amino acid composition of the investigated sequences [45]. Therefore, inferences about positive selection based on the dR/dC method should be treated with some caution.

## Results

**Figure 1:**
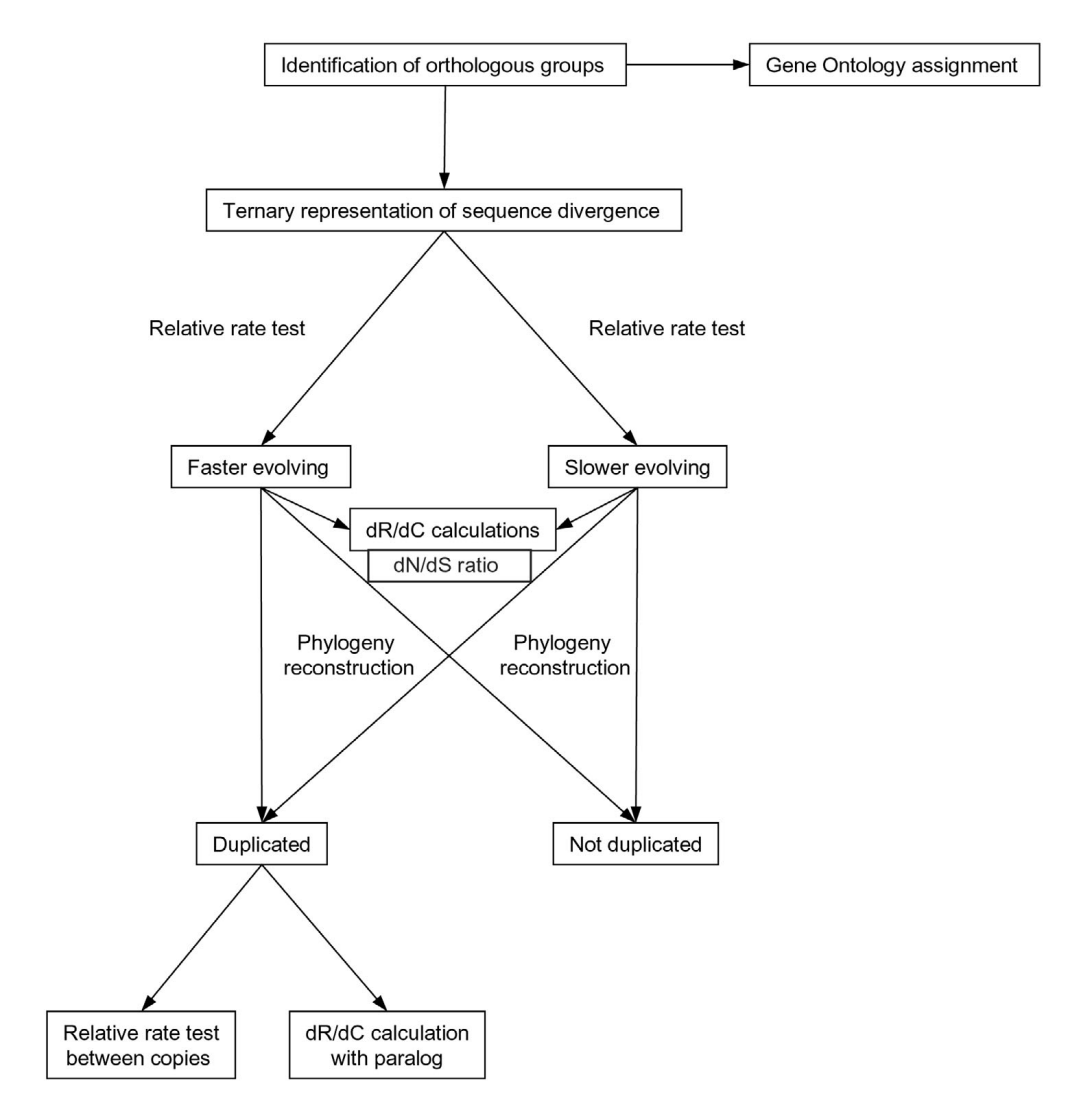
Flowchart of the analysis routine used in this study.

**Figure 2:**
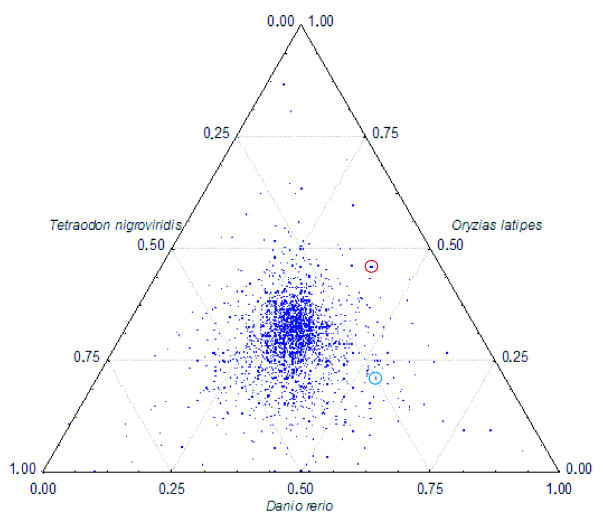
Example of a ternary representation of distances of fish species to human orthologs (*Danio rerio, Oryzias latipes, Tetraodon nigroviridis*). The red circle represents Lysyl-oxidase-like-1 (LOXL-1), a gene with significantly lower distance from *Tetraodon nigroviridis *to the human ortholog than to other fish species. The blue circle represents the ankyrin repeat domain 10, a gene with significantly higher distance from *Danio rerio *to the human ortholog than to other fish species.

**Figure 3:**
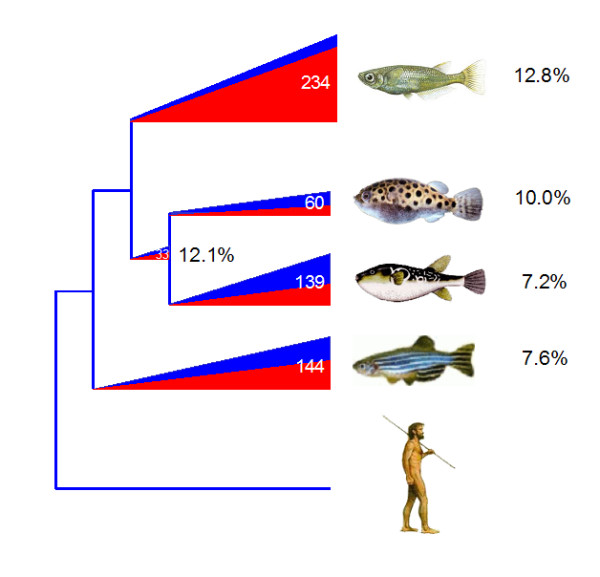
Phylogeny of the studied fish species. The proportion of genes with significantly lower (red) or higher (blue) distance to the human ortholog than to other fish species, are mapped onto the phylogeny as proportional triangles. Numbers within the triangle represent the total abundance of those genes. The percentages represent the corresponding proportion of transcription factors.

BLAST similarity searches of data sets of protein sequences of *Tetraodon nigroviridis *revealed a total of 12422 significant hits (e-value: 10^-50^) when compared to the human genome (outgroup). 12176 hits were found when comparing *Takifugu rubripes *to the human genome, 9619 hits were found for *Danio rerio *and 4681 hits were obtained for *Oryzias latipes*. For our comparative approach (Figure 1), 2466 orthology groups consisting of genes found in all four fish species could be assigned. Figure 2 shows an example of a ternary diagram of p-distances of *Tetraodon nigroviridis*, *Danio rerio *and *Oryzias latipes *amino acid sequences always with respect to the *Homo sapiens *genes. A total of 390 genes were found that have a smaller distance to the human ortholog than their orthologs in other fish species according to relative rate tests, whereas 220 genes show higher distances in one of the tested fish species' genomes (Table 1). In *Oryzias latipes *a higher percentage (8.43%) of genes show a smaller genetic distance to their human orthologs than in the other fish genomes. The number of genes with higher or lower distance for particular fish species is depicted in Figure 3. The majority of genes detected in *Oryzias latipes *and *Danio rerio *show smaller distances, whereas a higher amount of genes in both pufferfish species seem to have evolved faster (59%). All genes under positive Darwinian selection according to Hughes *et al*. [40] with asymmetric sequence divergence to the human ortholog are given for *Tetraodon nigroviridis *(Table 2), *Takifugu rubripes *(Table 3), *Danio rerio *(Table 4) and *Oryzias latipes *(Table 5). A complete list of the genes with divergent evolutionary rates from all fish species is provided in the supplementary material [see Supplementary File 1]. In all dN/dS calculations (data not shown) dS outperforms dN, which is expected given the old divergence times between the investigated species.

All the genes with divergent evolutionary rates have been tentatively annotated according to the best hit match according to the *Homo sapiens *UniGene data base. For these duplicates, the rate difference and the results of the relative rate tests between the two paralogs are given in the corresponding table (Table 2, 3, 4, 5). The annotation of the zebrafish best hits was additionally validated by comparisons to the ZFIN database (Table 4). Table 6 shows the number of fish specific paralogous pairs of genes discovered in this study with different evolutionary rates as compared to their human ortholog. Furthermore, the number of pairs where one paralog appears to have evolved under positive Darwinian selection is listed in Table 6. Accordingly, a total of 49 fish specific paralogous genes could be identified. Of these, 24 show a statistically significant accelerated rate of evolution in one of the two copies and 14 show positive Darwinian selection in one paralogous copy.

**Figure 4:**
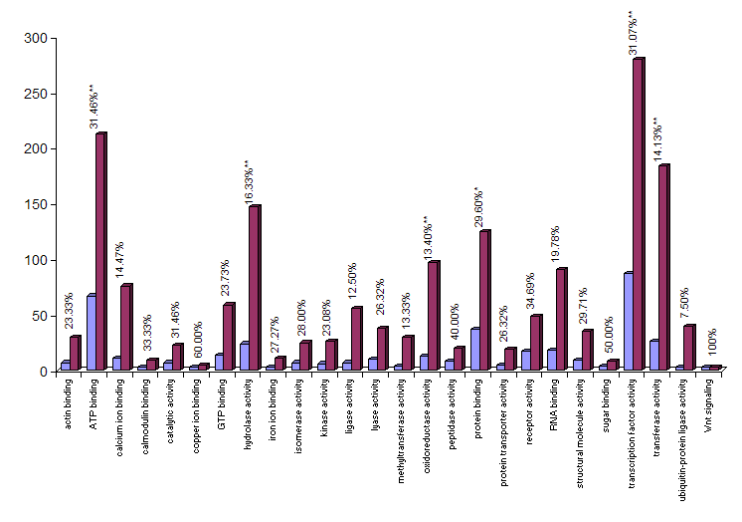
Abundances of gene functions (according to GO) of all fish protein genes with significantly lower or higher distance to the human ortholog than to other fish species (blue) compared to total abundances of gene functions (violet). The percentages represent the rates of the chosen genes compared to the total number of proteins of a given gene function. Significant differences between total and divergent gene abundances according to a χ^2^-Test are given at the 5% level with one asterisk and at the 1% level with two asterisks.

We then compared the relative frequency of genes of different gene functions according to the Gene Ontology (GO) classification. Figure 4 shows the number of genes with different functions for the complete dataset as well as for those genes where one of the fish species shows higher or smaller genetic distances. Some functions are likely to be overrepresented due to small sample sizes, like copper ion binding proteins, where 60% of the total numbers of proteins of this gene function show higher or lower distances in one fish species. This applies also to peptidases (40%), sugar binding proteins (60%) and members of the Wnt signaling pathway (100%). On the other hand, only one group (ubiquitin-protein ligases) shows a lower percentage (7.5%) than within the total number of proteins of this function in all comparisons (Figure 4).

### Overrepresentation of ATP-binding proteins and transcription factors

**Figure 5:**
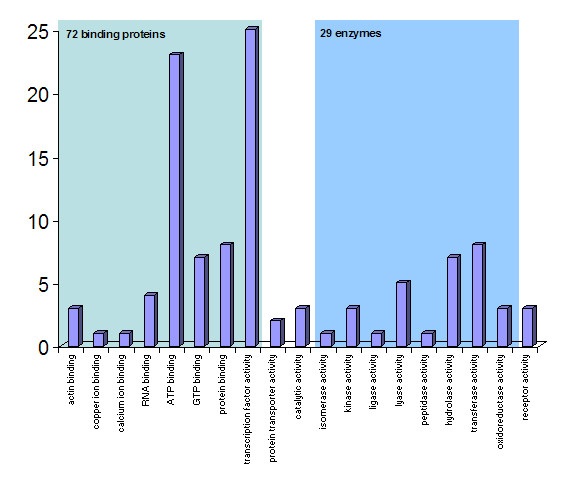
Abundances of gene functions (according to GO) of all fish protein genes under positive Darwinian selection with significantly lower or higher distance to the human ortholog than to other fish species.

**Figure 6:**
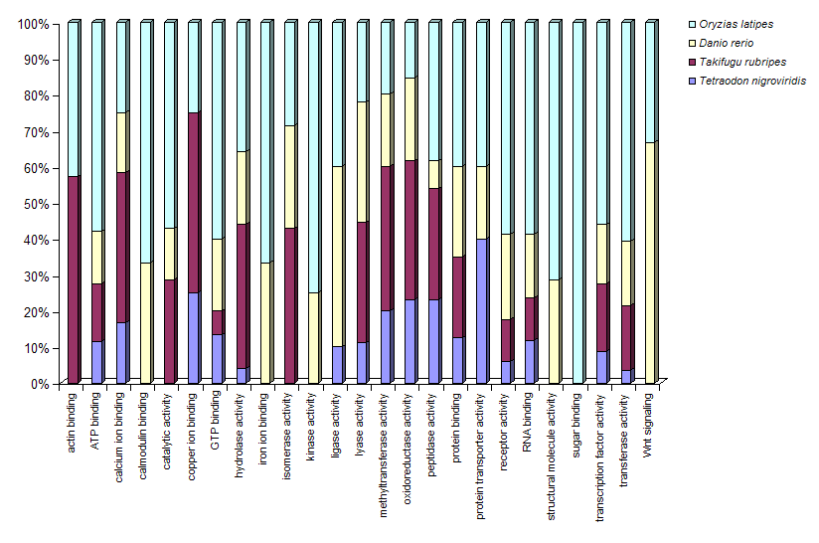
Comparison of rates of gene functions (according to GO) of all fish protein genes with significantly lower distance to the human ortholog than other fish species.

The relative frequency of genes of a certain functions of all fish genes under positive Darwinian selection (Figure 5) shows that more ATP binding proteins and transcription factors were found than could be expected based on the number of ATP binding proteins and transcription factors in general. A χ^2 ^(df = 2) test (depicted in Figure 4) confirmed that ATP binding proteins, hydrolases, oxidoreductases, transferases, and transcriptions factors occur significantly more often than could be expected by chance (highlighted by asterisks in Figure 4). The proportion of transcription factors detected in each species is on average similar (~10%) to other species (Figure 3). Overall, the majority of genes with divergent evolutionary rates among species are DNA-, ATP- and protein-binding proteins and enzymes (Figure 5). A comparison of the rates of Gene Ontology (GO) groups among the investigated fish genes is provided in Figure 6. Some GO groups include genes with divergent evolutionary rates only in zebrafish and medaka (calmodulin binding proteins, iron binding proteins, kinases, structural components, sugar binding proteins and members of the Wnt signaling pathway). In other GO groups the frequency of genes with divergent evolutionary rates from both pufferfish species is higher than 50% (e.g., calcium and copper ion binding proteins, methyltransferases, oxidoreductases and peptidases).

## Discussion

Our final data-set of genes that were recovered from all four species included a total of 2466 orthologous duplicates. This number was mainly limited by the small number of available *Oryzias latipes *sequences (4681). To examine whether these genes evolved at faster or slower evolutionary rates within one of the four fish species, relative rate tests were performed comparing each fish gene with the orthologs in other fish genomes. The human ortholog was used as outgroup. About two thirds of the 610 genes that showed significantly different evolutionary rates in one or more fish species with respect to the others showed a lower rate of molecular evolution for only one fish species (especially in *Oryzias latipes*). This implies that these genes are conserved in that particular lineage, most likely as a result of purifying selection (Figure 3). This high fraction of genes with a smaller genetic distance, i.e. slower evolutionary rate, might be due to the fact that comparisons based on BLAST searches are biased, so that genes with a higher rate are not recovered with the stringent e-value threshold we have applied.

The split between Sarcopterygii and Actinopterygii occurred about 400–450 million years ago [46]. Therefore it is possible that genes accumulated so many mutations and back mutations so that these genes could not be homologized applying very stringent BLAST conditions necessary for reliable annotations. However, we were able to identify 220 genes that show a statistically significant increased rate of molecular evolution. These genes presumably have been subjected to relaxed functional constraints. Genes, which did not accumulate more mutations than the average, are likely to have been subjected to purifying selection and thus, were not free to evolve. [43,47].

We found considerable numbers of genes under positive Darwinian selection according to Hughes *et al*. [40] with relaxed substitution rates at the amino acid level. It seems that in such cases selection acted more strongly to conserve the amino acid sequence of a gene in a particular lineage, possibly to maintain their ancestral function. In many cases, this function still seems to be shared with tetrapods and their common ancestors. Remarkably, 66% of the genes (Figure 5) that appear to have evolved under positive Darwinian selection turn out to be binding proteins, especially transcription factors and ATP binding proteins. Transcription factors represent on average ~10% of genes with significantly higher or lower evolutionary rates in each species, which is higher than the average proportion of transcription factors in vertebrate proteomes (~3%) (according to the EBI Eukaryotic Genome database). When identified as deviating gene, then it was in almost all cases because of a smaller rate of substitution, suggesting that these genes are highly conserved in at least one of the studied fish species. Mutations in a binding domain, as well as in regulatory regions [48,49], for example in a transcription factor, could negatively effect the expression of genes and therefore such mutations might be selected against.

None of the detected genes show evidence of positive selection using a dN/dS calculation following the method of Yang [50]. Due to the age of the investigated species, saturation might have blurred the signal of positive selection. However, a recent study [45] showed that the dR/dC ration is influenced by the transition/transversion ratio and amino acid composition of the investigated sequences. Therefore, inferences on positive selection based on this method should be treated cautiously.

A considerable high number of genes that have a higher or lower evolutionary rates are somehow related to neural development or functions of the brain. This might reflect differences in behavior and cognitive abilities between the investigated fish species.

We identified 49 new duplicated genes in this study, which had not been identified as such before, and which are likely to be the result of the fish-specific genome duplication [17,18,20,51]. Twenty-one of those show an increased rate in only one of the fish paralogs in a particular fish lineage. In 14 of these cases the increase in rate is likely to be the result of positive Darwinian selection (Table 6). Increases in the evolutionary rate in one copy could also be explained by the classical Ohno model of gene evolution [52], which predicts that one copy will evolve more rapidly at nonsynonymous sites compared to the other, as an effect of their redundancy. Nevertheless, it is difficult to demonstrate clear signs of positive Darwinian selection, when the duplication event is ancient [41]. For genes that show a faster evolutionary rate in one species and, in addition, evidence for positive Darwinian selection, one might expect concomitant divergence in function. On the other hand, for paralogs where positive Darwinian selection could not be demonstrated and where the evolutionary rates have not increased, one might assume that these genes have been under purifying selection or that these genes are about to lose their function according to the duplication-degeneration-complementation (DCC) model [30] due to the accumulation of degenerative mutations. Therefore, such genes may have a similar function or even be completely redundant functionally with respect to their ortholog. Although it might seem unlikely that two duplicates of one ancestral genes perform exactly the same function(s) after more than 300 million years of evolution [51,53], redundancy has been shown to be widespread in genomes of higher organisms [23-25].

### Tetraodon nigroviridis

Of the 93 genes with faster or slower evolutionary rates specific to *Tetraodon nigroviridis*, 40 were duplicated (43%). Unique increases in the rate of evolution for one copy of the duplicated genes, as a possible result of positive Darwinian selection, were most obvious in three cases (Table 2): The nuclear LIM interactor-interacting factor is an evolutionarily conserved transcriptional regulator that acts globally to silence neuronal genes [54]. The syntaxin binding protein 1 is a neural-specific, syntaxin-binding protein that may participate in the regulation of synaptic vesicle docking and fusion [55]. The hypothetical protein DKFZp564D0478 is known to be expressed in the hippocampus of vertebrates, although nothing is known about the function of this protein. Another positively selected duplicated gene is the ubiquitin domain containing 1 (UBTD1). There is no significant increase in the rate of evolution and it is only known that this protein is also expressed in the hippocampus [56]. Other genes are single-copy genes mainly coding for enzymes. However, the eukaryotic translation elongation factor I gamma gene encodes for a subunit of the elongation factor-1 complex, which is responsible for the delivery of aminoacyl tRNAs to the ribosome.

### Takifugu rubripes

A total of 172 genes could be identified, which show a faster or slower rate of molecular evolution in *Takifugu rubripes *as compared to the other four fish species. 74 (43%) of these genes are fish-specific duplicates. The total number is higher than in *Tetraodon*, which might reflect differences in sequence completeness. Positive Darwinian selection could be detected for 10% of the duplicated genes, most of which (70%) showed increased substitution rates in only one of the paralogs (Table 3): Syntaxin 3a is potentially involved in docking of synaptic vesicles at presynaptic active zones. In mammals, this gene occurs in different isoforms and is highly expressed in the larynx, which is evolutionarily and developmentally derived from branchial arches. The mitochondrial elongation factor G1 encodes one of the mitochondrial translation elongation factors [57]. The zinc finger protein 106 (ZFP106) is a conserved transcription factor of unknown function. However, its cDNA shares an extended region of identity with the *scr *homology domain 3 binding protein 3 (Sh3bp3) cDNA encoding a protein implicated in the insulin-signaling pathway [58]. In situ hybridization of mouse embryos confirmed that ZFP106 is predominantly expressed in tissues with high developmental activity of either nuclear respiratory factor-1 (brown fat and developing brain) or myogenin (striated muscle). Jumonji domain containing 2C is also a transcription factor containing PHD finger motifs [59]. PHD finger motifs are zinc finger-like sequences found in nuclear proteins that participate in chromatin-mediated transcriptional regulation and are present in a number of proto-oncogenes. The TNF receptor-associated protein 1 (TRAP1) is a chaperone belonging to the HSP90 family that expresses an ATPase activity [60]. Remarkably, TRAP1 interacts with the C-terminal ends of the proteins encoded by both exostosin 1 (EXT1) and exostosin 2 (EXT2). EXT1 has apparently also evolved under positive selection and was duplicated in both examined pufferfish species. Leprecan-like 1 is a cartilage-associated protein precursor found in articular chondrocytes and expressed in a variety of mammalian tissues [61].

Again the detected single copy genes are generally coding for a variety of enzymes. One exception is the protein encoded by *drebrin*-like. It is a cytoplasmic actin-binding protein thought to play a role in the process of neuronal growth and to be a member of the drebrin family of proteins that are developmentally regulated in the brain.

### Pufferfishes

Out of a total of 265 genes, 33 appeared to show an accelerated rate of evolution for both species This relatively high number is in concordance with the findings that, in the *Tetraodon *genome neutral nucleotide sequence evolution per year is about twice as fast as in humans [6,62]. Thus, it might be possible that the genome of pufferfish species evolves faster in general compared to other vertebrates, and possibly even other fishes. This could be linked to the processes related to the extreme degree of genome compaction in pufferfishes. In ten of the above mentioned cases we were able to detect signals of positive Darwinian selection (Tables 2 and 3).

One of the these 10 genes, the extracellular copper enzyme Lysyl-oxidase-like-1 (LOXL-1) initiates the cross linking of collagens and elastin in the process of building and deposition of elastic fibers. LOXL-1 thus seems to have an essential role in elastogenesis and resilience. LOXL-1 Mutant mice have among other problems a defect in elastic fiber renewal in adult tissues including the lower dermis of the skin [63]. It is tempting to speculate that the lower divergence of LOXL-1 in pufferfishes might be explained by the importance of elasticity of tissues in these species due to their ability to inflate as defense mechanism [64-67]. In addition to cross-linking extracellular matrix proteins, the encoded protein may have a role in tumor suppression [68]. Chloride intracellular channels are involved in chloride ion transport within various subcellular compartments. The chloride intracellular channel 5 gene (CLIC5) specifically associates with the cytoskeleton of placenta microvilli [69]. As mentioned before, one copy of EXT1 shows a higher rate of evolution due to positive Darwinian selection. EXT1 is a transferase involved in the chain elongation step of heparan sulfate biosynthesis. It appears to be a tumor suppressor [70].

Examples of positively selected single copy genes in both pufferfish species were 'twisted gastrulation' and TAFA2. 'Twisted gastrulation' encodes a secreted BMP-binding protein that is a BMP signalling agonist in the dorsal-ventral patterning pathway [71]. The TAFA proteins are predominantly expressed in specific regions of the brain, and are postulated to function as brain-specific chemokines or neurokines, that act as a regulator of immune and nervous cells [72].

### Danio rerio

Of the 144 genes with faster or slower evolutionary rates in *Danio rerio*, 38 belong to the group of duplicated genes. Among those genes with significantly higher rates in only one paralog (Table 4) are Cofilin 2, activin A type II receptor, Roundabout 1, GOT1, ARF4L, CBX3 and RAB25. Cofilin 2 controls reversibly actin polymerization and depolymerization in a ph-sensitive manner. It has the ability to bind g- and f-actin in a 1:1 ratio of cofilin to actin. It is the major component of intranuclear and cytoplasmic actin rods [73]. Activins are dimeric growth and differentiation factors, which belong to the transforming growth factor-beta (TGF-beta) superfamily of structurally related signaling proteins. Type II receptors are required for ligand-binding and for expression of type I receptors [74]. Roundabout 1 (ROBO1) encodes for an integral membrane protein that is both an axon guidance receptor and a cell adhesion receptor, and is involved in the decision by axons to cross the central nervous system midline [75]. Glutamic-oxaloacetic transaminase (GOT) is a pyridoxal phosphate-dependent enzyme, which exists in cytoplasmic and mitochondrial forms, GOT1 and GOT2. GOT plays a role in amino acid metabolism and the urea and tricarboxylic acid cycles. The two enzymes are homodimeric and show close homology [76]. The ADP-ribosylation factor 4-like is a member of the ADP-ribosylation factor family of GTP-binding proteins. ARF4L is closely similar to ARL4 and ARL7 and each has a nuclear localization signal and an unusually high guanine nucleotide exchange rate [77]. The protein encoded by the chromobox homolog 3 (CBX3) binds DNA and is a component of heterochromatin. This protein can also bind lamin B receptor, an integral membrane protein found in the inner nuclear membrane. The dual binding functions of the encoded protein may explain the association of heterochromatin with the inner nuclear membrane [78]. The gene encoding RAB25 may selectively regulate the apical recycling and/or transcytotic pathways [79]. Only little is known about the other genes detected in *Danio rerio*. Most of the single copy genes represent enzymes of varying function.

### Oryzias latipes

A total of 234 genes could be identified of genes with divergent evolutionary rates specific to *Oryzias latipes *when compared to the other fish species. Of those, only 14 were found to be duplicated. Only four genes with positive selected paralogs could be detected in *Oryzias latipes *(Table 5). One of those, with an accelerated rate of evolution in one of the paralogs is the ephrin receptor EphA2, which belongs to the ephrin receptor subfamily of the protein-tyrosine kinase family. EPH and EPH-related receptors have been implicated in mediating developmental events, particularly in the nervous system and limb development [80]. The ephrin receptors are divided into two groups based on the similarity of their extracellular domain sequences and their affinities for binding ephrin-A and ephrin-B ligands. This gene encodes a protein that binds ephrin-A ligands.

For two genes both paralogs show similar rates of evolution. The Endothelin-converting enzyme-1 is involved in the proteolytic processing of endothelin-1, -2, -3 to biologically active peptides [81]. Ficolin 1 encoded by FCN1 is predominantly expressed in the peripheral blood leukocytes, and has been postulated to function as a plasma protein with elastin-binding activity [82].

Other genes are single-copy genes mainly coding for enzymes with general functions. Remarkably, the number of positive selected genes in *Oryzias latipes *is higher than in the other investigated species and most of them have low substitution rates compared to other fish. Selection might thus act as maintenance for those genes and their function.

## Conclusion

We identified genes under positive Darwinian selection using a combination of BLAST searches and phylogenetic methods. With these methods we could also demonstrate that the measurement of positive selection is a good method to identify divergent fates of duplicated genes. Most genes behave differently in particular species, which implies that their function is somehow essential, and possibly of adaptive value, for the investigated fish species. We identified 49 of previously unknown duplicated genes where one of the paralogs is under positive Darwinian selection and shows a significantly high rate of molecular evolution whereas the other copy did not undergo such dramatic changes, most likely due to purifying selection. The fact that these duplicated genes show lineage specific evolutionary rates in the investigated fish species suggests that even after such a long time since the duplication event these genes might still contribute to lineage specific features. One might assume, that these genes therefore play a role in the diversification of lineages. Models such as the DDC model [30] might explain retention and functional divergence of anciently duplicated genes. It is also possible that neofunctionalisation of one paralog occurred. However, when subfunctionalisation is responsible for the functional divergence of genes, this is probably limited to differences in timing and tissue specificity of expression. It has also been suggested that a proportion of duplicate genes undergo rapid subfunctionalisation, accompanied by prolonged and substantial neofunctionalisation [31]. So far, there is little evidence that the new paralogs described here have completely novel functions. However, in several cases we could detect a significant increase in evolutionary rates in one of the duplicates. This is probably not due to relaxed functional constraints on the whole gene, but rather because duplicated genes experience a brief period of relaxed selection after duplication [26,41]. Duplicates that are being retained over longer evolutionary times are more likely to experience strong purifying selection.

## Methods

We used the database software EverEST 1.0 [83], which allows database searches on the basis of the BLAST algorithm and tests the association of results and phylogenetic analysis, making use of a relational database. Data sets of protein data from *Danio rerio *(Zebrafish Sequencing Group at the Sanger Institute, Zv4.0), EST data from *Oryzias latipes *(GenBank), protein data from *Takifugu rubripes *(JGI Fugu v3.0) and protein data from *Tetraodon nigroviridis *(GenBank) were screened against genome data from *Homo sapiens *(GenBank) using a translated BLAST routine (standard vertebrate code) with an expected value threshold of < 1 × 10^-50^. This relatively high value threshold was used in order to achieve high levels of confidence in the similarity searches, although such a stringent threshold might lead to the missing of some relevant orthologs. The query sequence and all best hits of every single search were aligned using the T-Coffee algorithm [84] implemented in EverEST. The automated analysis is depicted in Figure 1. Each sequence was tentatively assigned Gene Ontology (GO) classification based on annotation of the single 'best hit' match in BLASTX searches of *Homo sapiens *proteins (e≤ 10^-50^). Annotations described here are at the "inferred from electronic annotation" (IEA) level of evidence (The Gene Ontology Consortium 2001). Following the alignment, sequence divergence for every possible human-fish pair was estimated in EverEST as the observed proportion of amino acid sites at which the two sequences under comparison were different (Poisson correction). All alignment positions with gaps were excluded previously (complete deletion). This option is generally desirable because different regions of DNA or amino acid sequences often evolve under different evolutionary forces.

The distances (relative p-distances) were used to construct three-coordinate ternary representations (implemented in EverEST) for cross-species comparisons to visualize species-specific genes as outliers. A relative rate test was applied to each of the orthologous groups. We applied the nonparametric rate test developed by [39] and implemented in MEGA 3.0 [85], and compared the genes with their human and their fish orthologs. For orthologous groups, where the p-distance between *Homo sapiens *and fish amino acid sequences was significantly (p < 0.05) higher or smaller compared to the other three fish species, the ratio of the proportion of radical nonsynonymous difference (dR) per radical nonsynonymous site and the proportion of conservative nonsynonymous site (dC) was calculated using the program SCR3 [40]. This was done to evaluate the selective forces acting on those proteins. Conservative substitutions are amino acid replacements remain constant with respect to charge or polarity, while a substitution at a radical site involve amino acid replacements that changes charge or polarity [86]. In addition we conducted dN/dS calculations using the method by [50] implemented in PAML [87] for the 122 putative genes under positive selection according to dR/dC.

When BLAST identified one or more putative fish orthologs, protein sequences from all species were aligned using T-Coffee [84]. For each alignment, a preliminary tree was drawn. This tree facilitated the identification of identical sequences, sequences that varied only in length, and sequences within species that differed by few amino acids, all of which were removed from the alignment. Very similar sequences could be alleles at one locus or evidence of recent tandem duplications. In either case they were not likely to be important for our study of genome duplication. Phylogenies were reconstructed from the remaining sequences using Poisson-corrected genetic distances and the neighbor joining (NJ) algorithm [88] in MEGA 3.0 [85]. From these trees we identified sets of orthologous genes (i.e. genes which occurred only in monophyletic groups that matched the expected organismal topology). Regions where the alignment was unambiguous were retained and reanalyzed using NJ and maximum likelihood (ML) methods. For these last phylogenetic analyses the most closely related human paralogs (identified from the first NJ analyses) were used as outgroups. PHYML [89] was used to reconstruct ML. The best fitting models of sequence evolution for ML were obtained by ProtTest 1.2 [90].

To investigate whether or not one of the two fish paralogs evolved at a faster rate since their duplication, a relative rate test was applied to each of the genes. We applied the nonparametric rate test developed by [39] and implemented in MEGA 3.0 [85], and compared the paralogs with their human and another fish ortholog. The dR/dC ratio was calculated between the paralogs using the program SCR3 [40] to evaluate the selective forces acting on those.

## Authors' contributions

DS conceived the study, carried out the comparative analyses, and drafted the manuscript. WS participated in the design of the study, and helped to draft the manuscript. IB participated in the comparative analyses, the design of the study, and helped to draft the manuscript. AM participated in the study design and coordination and helped to draft the manuscript. All authors read and approved the final manuscript.

## Supplementary Material

Additional file 1 A complete list of genes with divergent evolutionary rates for all fish species of this study Click here for file

## Figures and Tables

**Table 1:** Abundance and proportion of protein genes of a given fish species with significantly lower or higher distance to the human ortholog than other species. The total number of comparisons was N = 2466. ^a ^denotes genes with significantly lower or higher distance for both pufferfish species

*Tetraodon nigroviridis*		*Takifugu rubripes*		*Danio rerio*		*Oryzias latipes*		Pufferfishes^a^	
lower	higher	lower	higher	lower	higher	lower	higher	lower	higher
25	35	58	81	80	64	208	26	19	14
∑ = 60		∑ = 139		∑ = 144		∑ = 234		∑ = 33	
1.01%	1.42%	2.35%	3.28%	3.24%	2.60%	8.43%	1.05%	0.77%	0.57%
∑ = 2.43%		∑ = 5.64%		∑ = 5.84%		∑ = 9.49%		∑ = 1.34%	

**Table 2:** *Tetraodon nigroviridis *protein genes under positive Darwinian selection with significantly lower (^a^) or higher (^b^) distance to the human ortholog than other fish species. Duplicated genes are given with rate differences between the two copies according to the relative rate test of Tajima (1993) at the 5% level with one asterisk and at the 1% level with two asterisks.

**GenBank Acc#**	**annotation according to human (UniGene)**	**duplicate rate difference**
CAG12160.1	lysyl oxidase-like 1 ^a^	0.247**
CAF89330.1	nuclear LIM interactor-interacting factor 2 ^a^	0.120**
CAG00079.1	chloride intracellular channel 5 ^a^	0.053*
CAG07128.1	twisted gastrulation ^a^	-
CAF96302.1	TAFA2 ^a^	0.014
CAF96493.1	serine/threonine protein kinase 6; aurora-A; IPL1-related kinase ^a^	0.067**
CAG00266.1	hypothetical protein DKFZp564D0478 ^a^	0.147*
CAF94681.1	eukaryotic translation elongation factor 1 gamma ^a^	-
CAF96262.1	TTK protein kinase ^a^	-
CAF91195.1	zinc finger protein 207 ^a^	-
CAF91040.1	exosome component 7^a^	-
CAF87120.1	hypothetical protein FLJ10996 ^a^	-
CAF92823.1	ubiquitin domain containing 1 ^a^	0.128
CAF91601.1	peroxisomal lon protease ^a^	-
CAF95463.1	hypothetical protein FLJ21156 ^a^	-
CAF91120.1	syntaxin binding protein 1 ^b^	0.350**
CAG08971.1	short-chain dehydrogenase/reductase ^b^	-
CAG13862.1	exostosin 1 ^b^	0.074

**Table 3:** *Takifugu rubripes *protein genes under positive Darwinian selection with significantly lower (^a^) or higher (^b^) distance to the human ortholog than other fish species. Duplicated genes are given with rate differences between the two copies according to the relative rate test of Tajima (1993) at the 5% level with one asterisk and at the 1% level with two asterisks.

**JGI Acc#**	**annotation according to human (UniGene)**	**duplicate rate difference**
FRUP00000147506	syntaxin 3A ^a^	0.178**
FRUP00000132184	lysyl oxidase-like 1 ^a^	0.207**
FRUP00000139282	chloride intracellular channel 5 ^a^	0.074
FRUP00000156028	twisted gastrulation ^a^	-
FRUP00000136434	mitochondrial elongation factor G1 ^a^	0.170*
FRUP00000139600	TAFA2 ^a^	-
FRUP00000129212	serine/threonine protein kinase 6 ^a^	-
FRUP00000153498	exostosin 1 ^a^	0.086
FRUP00000133663	zinc finger protein 106 homolog ^b^	0.201**
FRUP00000162759	hydroxysteroid (17-beta) dehydrogenase 4 ^a^	-
FRUP00000133083	dUTP pyrophosphatase ^a^	-
FRUP00000133686	excision repair cross-complementing 1 isoform 2 ^a^	-
FRUP00000132409	cytochrome c oxidase subunit Va ^a^	-
FRUP00000129950	glycine cleavage system protein H ^a^	-
FRUP00000140923	mitogen-activated protein kinase kinase kinase kinase 2 ^a^	-
FRUP00000157532	tryptophan rich basic protein ^a^	-
FRUP00000133839	stress-induced-phosphoprotein 1 ^a^	-
FRUP00000136741	dual specificity phosphatase 14 ^a^	-
FRUP00000148346	transitional epithelia response protein ^a^	-
FRUP00000149078	drebrin-like ^a^	-
FRUP00000151439	jumonji domain containing 2C ^a^	0.319**
FRUP00000134740	tumor necrosis factor type 1 receptor associated protein ^a^	0.099**
FRUP00000133087	leprecan-like 1 ^a^	0.317**
FRUP00000144902	calcium binding protein 5 ^a^	0.012
FRUP00000162608	neurogenic differentiation 4 ^a^	-
FRUP00000130779	FLJ21963 protein ^a^	-
FRUP00000143840	thioredoxin domain containing ^a^	-
FRUP00000154905	terminal deoxynucleotidyltransferase interacting factor 1 ^a^	-
FRUP00000128255	SMILE protein ^a^	-

**Table 4:** *Danio rerio *genes under positive Darwinian selection with significantly lower (^a^) or higher (^b^) distance to the human ortholog than other fish species. Duplicated genes are given with rate differences between the two copies according to the relative rate test of Tajima (1993) at the 5% level with one asterisk and at the 1% level with two asterisks. Known gene symbols according to ZFIN are also provided.

**Ensembl Acc#**	**annotation according to human (UniGene)**	**duplicate rate difference**	**ZFIN**
ENSDARP00000003385	ankyrin repeat domain 28 ^a^	0.285**	ankrd
ENSDARP00000024204	vesicle-associated membrane protein A isoform 2 ^a^	-	vapa
ENSDARP00000006783	cofilin 2 ^a^	0.078*	cfl2
ENSDARP00000026380	KIAA0073 protein ^a^	-	-
ENSDARP00000039499	haloacid dehalogenase-like hydrolase domain containing 2 ^a^	-	-
ENSDARP00000005886	arsenate resistance protein ARS2 isoform b ^a^	-	ars2
ENSDARP00000049098	thyroid hormone receptor interactor 13 ^a^	-	trip11
ENSDARP00000010419	DnaJ subfamily A member 2 ^a^	0.135	dnaja2
ENSDARP00000048880	gamma-glutamyl Carboxylase ^a^	-	ggcx
ENSDARP00000025803	SEC14 (S. cerevisiae)-like 1 ^a^	0.114	sec14l1
ENSDARP00000001039	hypothetical protein MGC10882 ^a^	-	-
ENSDARP00000038616	glutaryl-Coenzyme A dehydrogenase isoform a precursor ^a^	0.083	gcdh
ENSDARP00000024124	activin A type II receptor precursor ^a^	0.057**	acvr2a
ENSDARP00000025487	DKFZP434B168 ^a^	-	dkfzp434b168
ENSDARP00000024082	5-aminoimidazole-4-carboxamide ribonucleotide formyltransferase/IMP ^a^	-	atic
ENSDARP00000038219	roundabout 1 isoform a ^a^	0.276*	robo1
ENSDARP00000008117	HIRA interacting protein 5 ^a^	-	-
ENSDARP00000026984	beta catenin-like 1 ^a^	-	-
ENSDARP00000046992	carbohydrate (chondroitin) synthase 1 ^a^	-	chys^1^
ENSDARP00000016016	phosphatidylserine decarboxylase ^a^	-	-
ENSDARP00000023471	ADP-ribosylation factor-like 6 interacting protein ^a^	-	arl6ip
ENSDARP00000002978	aspartate aminotransferase 1 ^a^	0.015**	-
ENSDARP00000016111	ADP-ribosylation factor 4-like ^a^	0.032**	arl4
ENSDARP00000018086	androgen-induced 1 ^a^	-	aig1
ENSDARP00000015026	signal recognition particle 72 kDa ^a^	-	wu:fi03d11
ENSDARP00000038278	cytidine deaminase ^a^	-	aicda
ENSDARP00000006977	ankyrin repeat domain 10 ^b^	0.211	ankrd10
ENSDARP00000009789	chromobox homolog 3 HP1 gamma ^a^	0.009**	cbx
ENSDARP00000016540	RAB25 ^a^	0.170*	rab

**Table 5:** *Oryzias latipes *genes under positive Darwinian selection with significantly lower distance to the human ortholog than other fish species. Duplicated genes are given with rate differences between the two copies according to the relative rate test of Tajima (1993) at the 5% level with one asterisk and at the 1% level with two asterisks.

**GenBank Acc#**	**annotation according to human (UniGene)**	**duplicate rate difference**
AJ457222	DNA topoisomerase I	-
AU167343	CLIP-associating protein 1	-
AU167618	Ras-related associated with diabetes	-
AU167923	atrophin-1 interacting protein 1	-
AU176665	TAF6-like RNA polymerase II	-
AU177030	protein inhibitor of activated STAT X isoform beta	-
AU177176	metastasis suppressor 1	-
AU177627	Utrophin	-
AU180234	SNF2 histone linker PHD RING helicase	-
AV668786	AMPK-related protein kinase 5	-
AV670534	hippocampus abundant transcript 1	0.080*
BJ000375	ephrin receptor EphA2	0.149*
BJ003764	mitogen-activated protein kinase kinase kinase kinase 3	-
BJ004731	NICE-4 protein	-
BJ005724	tsJ homolog 1 isoform a	-
BJ007326	transcription factor T	-
BJ007726	G protein-coupled receptor 155	-
BJ008665	transducin-like enhancer protein 4	-
BJ008817	protein kinase C and casein kinase substrate in neurons 2	-
BJ021405	nucleoporin 62 kDa	-
BJ488812	fibulin 6	-
BJ490986	TRAF2 and NCK interacting kinase	-
BJ493563	PDZ domain containing ring finger 1	-
BJ495426	endothelin converting enzyme 1	0.084
BJ501370	DIP2-like protein isoform a	-
BJ517858	microfibrillar-associated protein 1	-
BJ519269	BCL2/adenovirus E1B 19kD interacting protein 2	-
BJ524171	component of oligomeric golgi complex 4	-
BJ527988	UDP-glucose ceramide glucosyltransferase-like 1	-
BJ539899	nuclear phosphoprotein PWP1	-
BJ543391	ribosomal protein L27a	-
BJ704447	pre-B-cell colony enhancing factor 1 isoform a	-
BJ704659	TCDD-inducible poly(ADP-ribose) polymerase	-
BJ706497	ubiquitin specific protease 34	-
BJ713400	neuro-oncological ventral antigen 1 isoform 2	-
BJ717464	thimet oligopeptidase 1	-
BJ724303	dihydrolipoamide S-succinyltransferase	-
BJ728080	myeloid/lymphoid or mixed-lineage leukemia 3	-
BJ728082	chromodomain helicase DNA binding protein 4	-
BJ728281	engulfment and cell motility 1 isoform 1	-
BJ728299	transcription factor AP-2 alpha	-
BJ728451	spermine synthase	-
BJ728665	Rb1-inducible coiled coil protein 1	-
BJ729946	intersectin 1 isoform ITSN-l	-
BJ730543	B-cell lymphoma 6 protein;	-
BJ730756	RE1-silencing transcription factor	-
BJ731205	MLL septin-like fusion	-
BJ733043	T-cell lymphoma invasion and metastasis 1	-
BJ733490	RAD21 homolog	-
BJ733549	SH3-domain binding protein 4	-
BJ734934	serine/threonine kinase 2	-
BJ735209	methylene tetrahydrofolate dehydrogenase 2 precursor	-
BJ735433	Huntingtin	-
BJ736888	bromodomain containing protein 2	-
BJ737071	fibrinogen C domain containing 1	0.061
BJ742416	JM1 protein	-
BJ743130	Moesin	-
BJ746379	microtubule associated serine/threonine kinase-like	-

**Table 6:** All fish specific paralogous pairs of proteins discovered in this study with significantly lower or higher distance to the human ortholog than other fish species. "dR/dC>1" represents the number of pairs where at least one paralog is under positive Darwinian selection according to the method of Hughes *et al*. (1990). "rel. rate sign." represents the number of pairs where at least one paralog shows a significantly higher rate of evolution according to the relative rate test of Tajima (1993).

	**dR/dC>1**		**rel. rate sign.**		**total number of pairs**
lower distance	13	44.83%	16	55.17%	29
higher distance	1	5.00%	8	40.00%	20
	
∑	14	28.57%	24	48.98%	49
