# Supplementary material for: Many genes in fish have species-specific asymmetric rates of molecular evolution
Source: BMC Genomics. 2006 Feb 8;7:20. doi: 10.1186/1471-2164-7-20 (PMC1413527; doi:10.1186/1471-2164-7-20)
Supplement: Additional file 1 — A complete list of genes with divergent evolutionary rates for all fish species of this study [file 1471-2164-7-20-S1.pdf]

Supplementary Information

S1: List of asymmetric divergent genes of *Tetraodon nigroviridis* with significantly lower distance to the human ortholog than other fish species.

GeneBank Acc#	annotation according to human (UniGene)
CAF87120	NP_061917 hypothetical protein FLJ10996
CAG14082	NP_036463 methionine sulfoxide reductase A; peptide met (O) reductase
CAF91195	NP_003448 zinc finger protein 207
CAF91040	NP_055819 exosome component 7
CAF91601	NP_113678 peroxisomal lon protease
CAF90334	NP_056132 pumilio homolog 2; pumilio (Drosphila) homolog 2
CAF95463	XP_371246 PREDICTED: hypothetical protein FLJ21156
CAF94681	NP_001395 eukaryotic translation elongation factor 1 gamma; elongation factor
CAF92823	NP_079230 ubiquitin domain containing 1
CAG01933	NP_003706 vesicle docking protein p115; transcytosis-associated protein
CAG01183	NP_001893 cystathionase isoform 1; homoserine deaminase; homoserine
CAG02866	NP_115920 kin of IRRE like 3
CAF96262	NP_003309 TTK protein kinase
CAG03725	NP_036272 solute carrier family 25 (mitochondrial carrier; dicarboxylate
CAG05711	NP_005725 homeodomain interacting protein kinase 3; homeodomain-interacting
CAG02777	NP_057036 RWD domain containing 1; PTD013 protein; CGI-24 protein
CAF98158	NP_054836 PRO0149 protein
CAG10808	NP_060705 CNDP dipeptidase 2 (metallopeptidase M20 family); cytosolic
CAF98538	NP_077284 hypothetical protein MGC4172
CAF98584	NP_068375 vacuolar protein sorting 11 (yeast homolog); vacuolar protein
CAG08659	NP_005739 YY1 associated factor 2
CAG08776	NP_078974 mitochondrial glutamate carrier 1
CAG08510	NP_116052 ubiquitin specific protease 30
CAG07399	NP_002323 low density lipoprotein-related protein 1; alpha-2-macroglobulin
CAG03960	NP_079423 hypothetical protein FLJ13448

S2: List of asymmetric divergent genes of *Tetraodon nigroviridis* with significantly higher distance to the human ortholog than other fish species.

GeneBank Acc#	annotation according to human (UniGene)
CAF92520	NP_055262 ADP-ribosylation factor interacting protein 1 (arfaptin 1)
CAF91538	XP_375074 PREDICTED: KIAA0391
CAF94283	NP_004143 ornithine decarboxylase antizyme 1; antizyme 1
CAF87029	NP_694558 hypothetical protein FLJ30596
CAF93255	NP_006463 thioredoxin interacting protein; upregulated by
CAF91349	NP_653321 multiple coiled-coil GABABR1-binding protein
CAF96622	NP_057178 COMM domain containing 2; HSPC042 protein
CAF95451	NP_001640 apical protein of Xenopus-like; APX homolog of Xenopus
CAF96104	NP_009200 syntaxin binding protein 3; syntaxin 4 binding protein
CAF95852	NP_110396 N-acetylneuraminate pyruvate lyase; dihydrodipicolinate synthase
CAG01934	NP_987100 Ras-GTPase activating protein SH3 domain-binding protein 2 isoform
CAG01161	NP_056099 KIAA0467 protein
CAF96336	NP_055512 C-terminal PDZ domain ligand of neuronal nitric oxide synthase
CAG05951	NP_000685 aldehyde dehydrogenase 3B1; aldehyde dehydrogenase 7
CAG05961	NP_002530 ornithine decarboxylase 1
CAG00619	NP_612451 hypothetical protein BC013949
CAG04864	NP_060284 hypothetical protein FLJ20422
CAG12701	XP_033371 PREDICTED: chromosome 14 open reading frame 120
CAG10304	NP_689601 hypothetical protein FLJ33282
CAG01778	NP_699193 hypothetical protein MGC46520; chromosome 6 open reading frame 158
CAG12647	XP_379784 PREDICTED: similar to GLCCI1 protein
CAG03482	NP_079415 hypothetical protein ARM
CAG13224	NP_056415 chromosome 20 open reading frame 22
CAF97249	NP_001955 early growth response 1; nerve growth factor-induced protein A
CAG10182	NP_000129 fibrillin 1
CAG07155	NP_060557 hypothetical protein FLJ10407
CAG09219	NP_000417 laminin alpha 2 subunit precursor; laminin M; merosin heavy chain
CAG08008	NP_057191 zinc finger RNA binding protein; M-phase phosphoprotein homolog
CAG08514	NP_066289 ubiquitin C
CAG06627	NP_004299 Rho GTPase activating protein 1; CDC42 GTPase-activating protein
CAG09658	NP_000104 torsin A
CAF97571	NP_620309 B-cell lymphoma 6 protein; B-cell CLL/lymphoma-6
CAG07575	NP_073585 tensin-like SH2 domain containing 1; tensin-like SH2
CAG05261	NP_001873 corticotropin releasing hormone binding protein; corticotropin
CAG10556	NP_064627 kinesin-like 7; kinesin-like protein 2

S3: List of asymmetric divergent genes of *Takifugu rubripes* with significantly lower distance to the human ortholog than other fish species.

JGI Acc#	annotation according to human (UniGene)
FRUP00000128255	NP_861448 SMILE protein
FRUP00000128906	NP_060528 cartilage acidic protein 1; chondrocyte expressed protein 68 kDa
FRUP00000129629	NP_001381 dystrobrevin alpha isoform 1; dystrophin-related protein 3
FRUP00000129698	NP_055686 ubiquitin specific protease 52; PABP-dependent poly(A) nuclease 2
FRUP00000129950	NP_004474 glycine cleavage system protein H (aminomethyl carrier)
FRUP00000130435	XP_043863 PREDICTED: similar to RIKEN cDNA 9930036E21 gene
FRUP00000130779	NP_078836 FLJ21963 protein
FRUP00000132018	NP_000062 cystathionine-beta-synthase; serine sulfhydrase; beta-thionase
FRUP00000132064	XP_376350 PREDICTED: Rap guanine nucleotide exchange factor (GEF) 2
FRUP00000132065	NP_006310 CDP-diacylglycerol--inositol 3-phosphatidyltransferase isoform 1
FRUP00000132409	NP_004246 cytochrome c oxidase subunit Va precursor; cytochrome c oxidase
FRUP00000133083	NP_001939 dUTP pyrophosphatase
FRUP00000133087	NP_060662 leprecan-like 1; myxoid liposarcoma associated protein 4
FRUP00000133159	NP_818932 erythrocyte membrane protein band 4.1-like 1 isoform b; neuron-type
FRUP00000133686	NP_001974 excision repair cross-complementing 1 isofrom 2
FRUP00000133839	NP_006810 stress-induced-phosphoprotein 1 (Hsp70/Hsp90-organizing protein)
FRUP00000134119	NP_060867 choline dehydrogenase
FRUP00000134740	NP_057376 tumor necrosis factor type 1 receptor associated protein
FRUP00000135019	NP_006693 neuropathy target esterase
FRUP00000136741	NP_008957 dual specificity phosphatase 14; MKP-1 like protein tyrosine
FRUP00000139307	NP_077002 hypothetical protein MGC955
FRUP00000140706	NP_009009 LIM domain binding 3; Z-band alternatively spliced PDZ-motif
FRUP00000140918	NP_803133 phosphatidic acid phosphatase type 2B; phosphatidic acid
FRUP00000140923	NP_004570 mitogen-activated protein kinase kinase kinase kinase 2; Rab8
FRUP00000142943	NP_001101 a disintegrin and metalloprotease domain 10
FRUP00000143454	NP_055045 signal recognition particle 68kDa
FRUP00000143706	NP_004499 isopentenyl-diphosphate delta isomerase; IPP isomerase
FRUP00000143840	NP_110382 thioredoxin domain containing; thioredoxin-related transmembrane
FRUP00000144209	NP_001327 cathepsin Z preproprotein; cathepsin X precursor; preprocathepsin
FRUP00000144902	NP_062829 calcium binding protein 5
FRUP00000145043	XP_375456 PREDICTED: hypothetical protein DKFZp761G2113
FRUP00000145071	NP_005947 methylenetetrahydrofolate dehydrogenase 1
FRUP00000146010	NP_006773 zinc finger protein-like 1; zinc-finger protein in MEN1 region
FRUP00000146416	NP_778231 juxtaposed with another zinc finger gene 1; TAK1-interacting
FRUP00000148287	XP_291222 PREDICTED: DKFZP586J0619 protein
FRUP00000148346	NP_037451 transitional epithelia response protein
FRUP00000149078	NP_054782 drebrin-like; src homology 3 domain-containing protein HIP-55
FRUP00000150535	NP_002221 junction plakoglobin; gamma-catenin; catenin
FRUP00000151439	NP_055876 jumonji domain containing 2C; gene amplified in squamous cell
FRUP00000151654	NP_001907 cytochrome c-1
FRUP00000152526	NP_001689 AU RNA-binding protein/enoyl-Coenzyme A hydratase precursor
FRUP00000154905	NP_443183 terminal deoxynucleotidyltransferase interacting factor 1
FRUP00000156102	NP_006322 C2f protein
FRUP00000156478	NP_116027 GATA binding protein 2; GATA-binding protein 2
FRUP00000157004	NP_004136 myosin IXB
FRUP00000157499	NP_733466 regulator of G-protein signalling 20; regulator of Gz-selective
FRUP00000157532	NP_004618 tryptophan rich basic protein; congenital heart disease 5 protein
FRUP00000159300	NP_065433 adenylate cyclase 2; ATP pyrophosphate-lyase
FRUP00000159951	NP_061326 13kDa differentiation-associated protein
FRUP00000160003	NP_009109 serine/threonine kinase receptor associated protein
FRUP00000161350	XP_496546 PREDICTED: kelch repeat and BTB (POZ) domain containing 9
FRUP00000161596	NP_987095 DEAD box polypeptide 42 protein; RNA helicase-like protein; SF3b125
FRUP00000161887	NP_004126 isocitrate dehydrogenase 3 (NAD+) gamma isoform a precursor
FRUP00000162556	NP_060739 membrane-type 1 matrix metalloproteinase cytoplasmic tail binding
FRUP00000162595	NP_112179 FIP1-like 1; rearranged in hypereosinophilia
FRUP00000162608	NP_067014 neurogenic differentiation 4
FRUP00000162759	NP_000405 hydroxysteroid (17-beta) dehydrogenase 4
FRUP00000165169	NP_775904 hypothetical protein FLJ90805

S4: List of asymmetric divergent genes of *Takifugu rubripes* with significantly higher distance to the human ortholog than other fish species.

JGI Acc#	annotation according to human (UniGene)
FRUP00000144178	NP_000051 biotinidase precursor
FRUP00000134369	NP_000084 alpha 1 type V collagen preproprotein
FRUP00000128173	NP_000087 ceruloplasmin (ferroxidase)
FRUP00000165521	NP_000215 keratin 18; cytokeratin 18
FRUP00000127640	NP_000387 cathepsin K preproprotein; cathepsin X; cathepsin O1; cathepsin O2
FRUP00000129867	NP_000703 biliverdin reductase A
FRUP00000148572	NP_000860 5-hydroxytryptamine (serotonin) receptor 3A isoform b precursor
FRUP00000158231	NP_001232 cyclin T2 isoform a; cyclin T2a; cyclin T2b; SDS-stable
FRUP00000158159	NP_001238 ectonucleoside triphosphate diphosphohydrolase 6; CD39-like 2
FRUP00000139774	NP_001334 disabled homolog 2; mitogen-responsive phosphoprotein
FRUP00000162226	NP_001601 lysosomal acid phosphatase 2 precursor
FRUP00000143645	NP_001695 brain-specific angiogenesis inhibitor 3
FRUP00000139096	NP_002151 tenascin C (hexabrachion); Hexabrachion (tenascin)
FRUP00000153929	NP_002328 low density lipoprotein receptor-related protein associated protein
FRUP00000131103	NP_002395 microfibrillar-associated protein 4; microfibril-associated
FRUP00000161646	NP_002791 proteasome beta 9 subunit isoform 1 proprotein; proteasome-related
FRUP00000151511	NP_003400 zinc finger protein 161 homolog
FRUP00000151465	NP_003661 differentiated embryo chondrocyte expressed gene 1
FRUP00000135860	NP_004070 cathepsin S preproprotein
FRUP00000144318	NP_004384 dystroglycan 1 precursor; alpha-dystroglycan; Dystrophin-associated
FRUP00000132337	NP_004534 nebulin
FRUP00000155152	NP_005202 colony stimulating factor 1 receptor precursor; CD115 antigen; FMS
FRUP00000138618	NP_005214 deoxyribonuclease I
FRUP00000159393	NP_005224 ephrin receptor EphA3 isoform a precursor; eph-like tyrosine kinase
FRUP00000150543	NP_005236 FAT gene product
FRUP00000159806	NP_005633 TATA box-binding protein-associated factor 2F; TAF7 RNA polymerase
FRUP00000154451	NP_005873 macrophage erythroblast attacher; erythroblast macrophage protein
FRUP00000143167	NP_006258 RAN binding protein 2; nucleoporin 358; nuclear pore complex protein
FRUP00000143799	NP_006658 progesterone receptor membrane component 1; progesterone binding
FRUP00000157168	NP_006735 RBP4 gene product
FRUP00000132356	NP_006801 for protein disulfide isomerase-related
FRUP00000158136	NP_006843 tousled-like kinase 2; serine/threonine kinase; tousled-like kinase
FRUP00000156726	NP_009111 trehalase (brush-border membrane glycoprotein)
FRUP00000144075	NP_009135 DEAD (Asp-Glu-Ala-Asp) box polypeptide 20; DEAD-box protein DP103
FRUP00000164640	NP_037452 host cell factor C2; host cell factor 2
FRUP00000143675	NP_054861 nudix-type motif 5; nucleoside diphosphate linked moiety X-type
FRUP00000161258	NP_055227 calcyclin binding protein
FRUP00000138226	NP_055291 PDZ and LIM domain 3; alpha-actinin-2-associated LIM protein
FRUP00000165229	NP_055472 KIAA0406 gene product
FRUP00000140193	NP_055530 Rho GTPase-activating protein; RhoGAP involved in the
FRUP00000156607	NP_055736 latrophilin 1; lectomedin-2
FRUP00000128998	NP_056014 ankyrin repeat domain 28
FRUP00000130545	NP_057001 glutathione transferase kappa 1; glutathione S-transferase subunit
FRUP00000160056	NP_057019 CGI-01 protein isoform 1
FRUP00000133120	NP_057216 myelin gene expression factor 2
FRUP00000148750	NP_057226 steroid dehydrogenase homolog; 3-ketoacyl-CoA reductase
FRUP00000146140	NP_057307 protein kinase C and casein kinase substrate in neurons 3
FRUP00000150117	NP_057569 chromosome 6 open reading frame 55; My012 protein
FRUP00000159307	NP_060480 estrogen-related receptor beta like 1; HIP1 protein interactor
FRUP00000152190	NP_060833 chromosome 10 open reading frame 59
FRUP00000164001	NP_060857 spermatid perinuclear RNA-binding protein
FRUP00000141350	NP_060863 hypothetical protein FLJ11336
FRUP00000156924	NP_060898 hepatocellular carcinoma-associated antigen 66
FRUP00000159600	NP_061917 hypothetical protein FLJ10996
FRUP00000129889	NP_061921 HCV NS3-transactivated protein 1
FRUP00000143684	NP_064507 potassium channel modulatory factor 1
FRUP00000129554	NP_064555 nicalin; nicastrin-like protein
FRUP00000158899	NP_066928 phospholipid scramblase 1
FRUP00000138347	NP_067045 TcD37 homolog; prune
FRUP00000147503	NP_071330 differentially expressed in FDCP 6 homolog; IRF4-binding protein
FRUP00000146283	NP_071350 chromosome 14 open reading frame 133
FRUP00000146080	NP_110378 transcription factor 8 (represses interleukin 2 expression)
FRUP00000161888	NP_112174 tripartite motif-containing 8; ring finger protein 27
FRUP00000149115	NP_112573 HMG-box transcription factor TCF-3
FRUP00000135046	NP_542400 hypothetical protein MGC9564
FRUP00000143016	NP_660202 similar to CG3714 gene product
FRUP00000139310	NP_660348 mitochondrial solute carrier protein
FRUP00000147385	NP_689584 hypothetical protein MGC26818
FRUP00000143171	NP_689742 G protein-coupled receptor 155
FRUP00000150330	NP_776250 zinc transporter ZnT-8
FRUP00000147595	NP_892017 interferon regulatory factor 2 binding protein 2
FRUP00000140238	XP_085151 PREDICTED: YLP motif containing 1
FRUP00000150434	XP_114303 PREDICTED: FERM domain containing 4B
FRUP00000147544	XP_370618 PREDICTED: hypothetical protein FLJ20294
FRUP00000131153	XP_372038 PREDICTED: hypothetical protein FLJ32731
FRUP00000154528	XP_374996 PREDICTED: AMPK-related protein kinase 5
FRUP00000143731	XP_376525 PREDICTED: zinc finger and BTB domain containing 24
FRUP00000127095	XP_376652 PREDICTED: distal-less homeo box 6

S5: List of asymmetric divergent genes of *Tetraodon nigroviridis* and *Takifugu rubripes* with significantly lower distance to the human ortholog than other fish species.

JGI Acc#	GeneBank Acc#	annotation according to human (UniGene)
FRUP00000136080	CAG12906	NP_000118 exostosin 1
FRUP00000147506	CAG04790	NP_004168 syntaxin 3A
FRUP00000132184	CAG12160	NP_005567 lysyl oxidase-like 1
FRUP00000136563	CAF89330	NP_005721 nuclear LIM interactor-interacting factor 2
FRUP00000163237	CAF90351	NP_006708 spindlin
FRUP00000127131	CAF99488	NP_009049 triple functional domain (PTPRF interacting)
FRUP00000139282	CAG00079	NP_058625 chloride intracellular channel 5
FRUP00000150654	CAF90397	NP_060113 mbt domain containing 1
FRUP00000137831	CAF91044	NP_062825 fibroblast growth factor 20
FRUP00000156028	CAG07128	NP_065699 twisted gastrulation
FRUP00000130264	CAG08921	NP_065842 serine/threonine protein kinase TAO1 homolog; STE20-like kinase
FRUP00000151490	CAF96898	NP_065924 leucine rich repeat neuronal 1
FRUP00000141647	CAF97238	NP_068579 SEC8 protein; exocyst complex component 4; secretory protein SEC8
FRUP00000136434	CAF97420	NP_079272 mitochondrial elongation factor G1; elongation factor G1
FRUP00000130105	CAF95949	NP_079351 hypothetical protein FLJ23445
FRUP00000162856	CAG11714	NP_110426 golgi membrane protein SB140; smooth muscle cell associated protein
FRUP00000145525	CAG00266	NP_115501 hypothetical protein DKFZp564D0478
FRUP00000139600	CAF96302	NP_848634 TAFA2
FRUP00000129212	CAF96493	NP_940839 serine/threonine protein kinase 6; aurora-A; IPL1-related kinase

S6: List of asymmetric divergent genes of *Tetraodon nigroviridis* and *Takifugu rubripes* with significantly higher distance to the human ortholog than other fish species.

JGI Acc#	GeneBank Acc#	annotation according to human (UniGene)
FRUP00000133413	CAG11348	NP_001989 fibulin 2 precursor; Fibulin-2
FRUP00000146417	CAF99414	NP_006015 Tax1 (human T-cell leukemia virus type I) binding protein 1
FRUP00000163281	CAF92734	NP_006604 GRB2-related adaptor protein; growth factor receptor-bound protein
FRUP00000146457	CAF90574	NP_054727 JM1 protein
FRUP00000131671	CAG06859	NP_055816 SMART/HDAC1 associated repressor protein; Msx2 interacting nuclear
FRUP00000159381	CAG00591	NP_062552 hypothetical protein FLJ20257
FRUP00000153117	CAG10911	NP_064714 pappalysin 2 isoform 1; pregnancy-associated plasma preproprotein-A2
FRUP00000138347	CAF90087	NP_067045 TcD37 homolog; prune
FRUP00000158273	CAG01232	NP_071918 zinc finger protein 106 homolog; zinc finger protein 106 homolog
FRUP00000132611	CAG10551	NP_071940 chromosome 14 open reading frame 135; F protein-binding protein 2
FRUP00000138063	CAG05824	NP_078824 hypothetical protein FLJ23047
FRUP00000131405	CAG06666	NP_078857 chromosome 6 open reading frame 60
FRUP00000144899	CAF88516	NP_116219 cirhin; testis expressed gene 292
FRUP00000161684	CAG10926	NP_777596 proprotein convertase subtilisin/kexin type 9 preproprotein; neural

S7: List of asymmetric divergent genes of *Danio rerio* with significantly lower distance to the human ortholog than other fish species.

Ensembl Acc#	annotation according to human (UniGene)
ENSDARP00000000426	NP_061845 ganglioside-induced differentiation-associated protein 1
ENSDARP00000001039	NP_116062 hypothetical protein MGC10882
ENSDARP00000002077	NP_775740 hypothetical protein LOC92912
ENSDARP00000002088	NP_036347 meningioma expressed antigen 5 (hyaluronidase)
ENSDARP00000002978	NP_002070 aspartate aminotransferase 1
ENSDARP00000003385	NP_056014 ankyrin repeat domain 28
ENSDARP00000003460	NP_113671 itchy homolog E3 ubiquitin protein ligase; atrophin-1 interacting
ENSDARP00000005886	NP_877952 arsenate resistance protein ARS2 isoform b
ENSDARP00000006171	NP_004459 four and a half LIM domains 3
ENSDARP00000006783	NP_619579 cofilin 2
ENSDARP00000007120	NP_060793 phosphatidylinositol 4-kinase type-II beta
ENSDARP00000007480	NP_064627 kinesin-like 7; kinesin-like protein 2
ENSDARP00000007485	NP_003913 guanine nucleotide exchange factor p532
ENSDARP00000008117	NP_056515 HIRA interacting protein 5; iron-sulfur cluster scaffold protein
ENSDARP00000008926	NP_061120 MAP/microtubule affinity-regulating kinase 1
ENSDARP00000009000	NP_002754 prospero-related homeobox 1
ENSDARP00000009304	NP_004795 WD40 protein Ciao1
ENSDARP00000009789	NP_057671 chromobox homolog 3; heterochromatin protein HP1 gamma; HP1 gamma
ENSDARP00000010282	NP_004090 stomatin isoform a; erythrocyte membrane protein band 7.2
ENSDARP00000010419	NP_005871 DnaJ subfamily A member 2; cell cycle progression 3 protein; HIRA
ENSDARP00000012263	NP_054762 DKFZP564O123 protein
ENSDARP00000014083	NP_079011 F-box protein 31; putative breast cancer tumor-suppressor; MGC15419
ENSDARP00000014426	NP_005332 GLI-Kruppel family member HKR3
ENSDARP00000014918	NP_004512 kinesin family member 5B; kinesin 1 (110-120kD)
ENSDARP00000015026	NP_008878 signal recognition particle 72kDa
ENSDARP00000015333	NP_940965 Similar to RIKEN cDNA 1810038N08 gene
ENSDARP00000016016	NP_055153 phosphatidylserine decarboxylase
ENSDARP00000016111	NP_001652 ADP-ribosylation factor 4-like; ADP-ribosylation factor-like 6
ENSDARP00000016540	NP_065120 RAB25
ENSDARP00000017570	NP_055467 importin 13; Ran binding protein 13; karyopherin 13
ENSDARP00000017756	NP_620482 myosin IIIB
ENSDARP00000017919	NP_075391 FLJ21919 protein
ENSDARP00000018086	NP_057192 androgen-induced 1; androgen induced protein; CGI-103 protein
ENSDARP00000020960	NP_057121 CGI-94 protein
ENSDARP00000021263	NP_005069 transducin-like enhancer protein 3; transducin-like enhancer of
ENSDARP00000022353	NP_055055 LIM domains containing 1
ENSDARP00000022377	NP_079532 HLA-B associated transcript 8 BAT8 isoform b; G9A histone
ENSDARP00000023471	NP_055976 ADP-ribosylation factor-like 6 interacting protein
ENSDARP00000023764	NP_006383 nucleolar protein 5A; nucleolar protein 5A (56kD with KKE/D repeat)
ENSDARP00000023807	NP_055268 putative breast adenocarcinoma marker
ENSDARP00000023828	NP_115834 PHD-like zinc finger protein
ENSDARP00000024082	NP_004035 5-aminoimidazole-4-carboxamide ribonucleotide formyltransferase/IMP
ENSDARP00000024124	NP_001607 activin A type II receptor precursor
ENSDARP00000024204	NP_919415 vesicle-associated membrane protein-associated protein A isoform 2
ENSDARP00000024552	NP_002121 3-hydroxy-3-methylglutaryl-Coenzyme A synthase 1 (soluble
ENSDARP00000025226	NP_002789 proteasome beta 6 subunit; proteasome subunit Y; proteasome subunit
ENSDARP00000025370	NP_005708 actin related protein 2/3 complex subunit 5; Arp2/3 protein complex
ENSDARP00000025487	NP_056249 DKFZP434B168 protein
ENSDARP00000025803	NP_002994 SEC14 (S. cerevisiae)-like 1
ENSDARP00000026312	NP_001454 frizzled-related protein; Fritz; Frzb-1; fre; frizzled (Drosophila)
ENSDARP00000026380	NP_056157 KIAA0073 protein
ENSDARP00000026984	NP_110517 beta catenin-like 1; chromosome 20 open reading frame 33
ENSDARP00000027703	NP_065806 tumor differentially expressed 2
ENSDARP00000033448	NP_005771 lipoma HMGIC fusion partner
ENSDARP00000034972	NP_003861 IQ motif containing GTPase activating protein 1; rasGAP-like with IQ
ENSDARP00000035170	NP_113619 component of golgi transport complex 3; tethering factor SEC34
ENSDARP00000035342	XP_035825 PREDICTED: KIAA0143 protein
ENSDARP00000035845	NP_056415 chromosome 20 open reading frame 22
ENSDARP00000035932	NP_859074 hypothetical protein LOC286097
ENSDARP00000037454	NP_849163 ring finger protein 166; hypothetical zinc finger protein MGC2647
ENSDARP00000038219	NP_002932 roundabout 1 isoform a; roundabout 1; axon guidance receptor
ENSDARP00000038278	NP_001776 cytidine deaminase
ENSDARP00000038616	NP_000150 glutaryl-Coenzyme A dehydrogenase isoform a precursor
ENSDARP00000038907	NP_005907 minichromosome maintenance protein 7 isoform 1; DNA replication
ENSDARP00000039044	NP_056170 joined to JAZF1
ENSDARP00000039499	NP_115500 haloacid dehalogenase-like hydrolase domain containing 2
ENSDARP00000041136	NP_002071 aspartate aminotransferase 2 precursor
ENSDARP00000041858	NP_065752 KIAA1160 protein
ENSDARP00000043210	NP_055776 rap2 interacting protein x
ENSDARP00000043512	XP_371074 PREDICTED: putative ankyrin-repeat containing protein
ENSDARP00000044308	NP_510965 fuse-binding protein-interacting repressor isoform a; siah binding
ENSDARP00000045797	NP_631895 adenosine monophosphate deaminase 2 (isoform L)
ENSDARP00000046992	NP_055733 carbohydrate (chondroitin) synthase 1; chondroitin synthase
ENSDARP00000047218	NP_001625 S-adenosylmethionine decarboxylase 1 precursor
ENSDARP00000047608	NP_006244 AMP-activated protein kinase beta 1 non-catalytic subunit
ENSDARP00000047722	NP_742067 similar to F10G7.10.p
ENSDARP00000048716	NP_036396 chromosome 22 open reading frame 5
ENSDARP00000048880	NP_000812 gamma-glutamyl carboxylase
ENSDARP00000049098	NP_004228 thyroid hormone receptor interactor 13; thyroid receptor
ENSDARP00000049611	NP_009128 frizzled 10; frizzled 10 precursor; frizzled (Drosophila) homolog

S8: List of asymmetric divergent genes of *Danio rer*io with significantly higher distance to the human ortholog than other fish species.

Ensembl Acc#	annotation according to human (UniGene)
ENSDARP00000044235	NP_612403 hypothetical protein BC008217
ENSDARP00000005405	NP_061940 hypothetical protein FLJ10613
ENSDARP00000022032	NP_689656 retinol dehydrogenase 12 (all-trans and 9-cis)
ENSDARP00000017530	NP_112225 ring finger protein 146; 2610509H23Rik; dactylidin
ENSDARP00000049133	NP_076983 hypothetical protein MGC3162
ENSDARP00000043042	NP_065803 G protein-coupled receptor 158
ENSDARP00000012594	NP_056183 pannexin 1; MRS1 protein; innexin
ENSDARP00000042393	NP_057165 palladin; CGI-151 protein
ENSDARP00000028345	NP_037511 dipeptidyl peptidase 7 preproprotein; quiescent cell proline
ENSDARP00000046430	NP_000405 hydroxysteroid (17-beta) dehydrogenase 4
ENSDARP00000036344	NP_056495 chromosome 2 open reading frame 24
ENSDARP00000050435	NP_110382 thioredoxin domain containing; thioredoxin-related transmembrane
ENSDARP00000034339	NP_005345 jun D proto-oncogene; transcription factor jun-D; JunD-FL isoform
ENSDARP00000013013	NP_631918 KCCR13L
ENSDARP00000006977	NP_060134 ankyrin repeat domain 10
ENSDARP00000012660	NP_079131 ARP5 actin-related protein 5 homolog; ARP5 (actin-related protein)
ENSDARP00000025744	XP_097886 PREDICTED: hypothetical protein XP_097886
ENSDARP00000030663	NP_056311 DKFZP434I116 protein isoform 1
ENSDARP00000049468	NP_004963 Janus kinase 2; tyrosine-protein kinase JAK2
ENSDARP00000046025	NP_000497 coagulation factor II precursor; prothrombin
ENSDARP00000014549	NP_000539 tuberous sclerosis 2 isoform 1; tuberin isoform 1; tuberin isoform 2
ENSDARP00000038928	NP_006253 peripherin; neurofilament 4 (57kD)
ENSDARP00000006884	NP_004514 kinesin family member 11; thyroid receptor interacting protein 5
ENSDARP00000035334	NP_001631 N-acylaminoacyl-peptide hydrolase; acylaminoacyl-peptidase
ENSDARP00000009932	NP_002609 peroxisome biogenesis factor 13
ENSDARP00000040085	NP_009160 protein kinase C and casein kinase substrate in neurons 2; pacsin 2
ENSDARP00000004933	NP_079105 hypothetical protein FLJ22662
ENSDARP00000041984	NP_056517 chromosome 2 open reading frame 25
ENSDARP00000048867	NP_055769 rabphilin 3A homolog
ENSDARP00000018313	NP_004321 BCL2/adenovirus E1B 19kD interacting protein 2; BCL2/adenovirus E1B
ENSDARP00000006061	NP_079228 chromosome 14 open reading frame 160
ENSDARP00000023896	NP_733821 lamin A/C isoform 1 precursor; 70 kDa lamin; progeria 1
ENSDARP00000018746	NP_060343 ankyrin repeat and SOCS box-containing 6 isoform 1
ENSDARP00000023886	NP_056416 hect domain and RLD 4
ENSDARP00000025759	NP_005782 M-phase phosphoprotein 10
ENSDARP00000004443	NP_003309 TTK protein kinase
ENSDARP00000045818	XP_291947 PREDICTED: similar to hephaestin
ENSDARP00000026009	NP_071435 PP1201 protein
ENSDARP00000004123	NP_001074 phosphodiesterase 5A isoform 1; cGMP-binding cGMP-specific
ENSDARP00000049572	NP_005248 GATA binding protein 6; GATA-binding protein 6
ENSDARP00000034390	NP_079425 hypothetical protein FLJ20920
ENSDARP00000016765	NP_005300 glutamic-pyruvate transaminase (alanine aminotransferase)
ENSDARP00000007458	NP_874365 scribble isoform a
ENSDARP00000041441	NP_001135 autocrine motility factor receptor isoform a
ENSDARP00000003579	NP_006304 ubiquitin specific protease 15; deubiquitinating enzyme
ENSDARP00000046868	NP_858045 nuclear receptor coactivator 3 isoform a; amplified in breast
ENSDARP00000038702	NP_115920 kin of IRRE like 3
ENSDARP00000046172	NP_079100 nuclear protein UKp68 isoform 1
ENSDARP00000009626	NP_006803 amplified in osteosarcoma
ENSDARP00000050527	NP_000541 tyrosinase-related protein 1
ENSDARP00000046451	NP_079107 PRIP-interacting protein PIPMT; PRIP-interacting protein
ENSDARP00000034236	NP_055876 jumonji domain containing 2C; gene amplified in squamous cell
ENSDARP00000025891	NP_002499 nidogen (enactin); Nidogen; nidogen (entactin)
ENSDARP00000004251	NP_653185 hypothetical protein FLJ30525
ENSDARP00000003415	NP_068775 glutamate receptor 6 isoform 1 precursor; excitatory amino acid
ENSDARP00000019313	NP_001837 alpha 2 type IV collagen preproprotein; canstatin
ENSDARP00000023514	NP_060573 leucine rich repeat containing 5; leucine-rich repeat-containing 5
ENSDARP00000018351	NP_861454 collomin
ENSDARP00000021392	NP_079123 transmembrane channel-like 7
ENSDARP00000011386	NP_055203 hypothetical protein MGC29875
ENSDARP00000046347	NP_005551 laminin alpha 5; laminin alpha-5 chain
ENSDARP00000003022	NP_061335 kelch-like 9
ENSDARP00000048028	NP_071407 cadherin related 23 isoform 1 precursor; cadherin-23; otocadherin
ENSDARP00000049495	NP_077006 melanophilin

S9: List of asymmetric divergent genes of *Oryzias latipes* with significantly lower distance to the human ortholog than other fish species.

GeneBank Acc#	annotation according to human (UniGene)
AU167144	NP_003394 YY1 transcription factor
AU167274	NP_000156 connexin 43; oculodentodigital dysplasia (syndactyly type III)
AU167343	NP_056097 CLIP-associating protein 1; multiple asters 1
AU167618	NP_004156 Ras-related associated with diabetes; RAS (RAD and GEM) like GTP
AU167785	NP_055741 slit and trk like 3 protein; slit and trk like gene 3
AU167800	NP_065759 solute carrier family 12 member 5; erythroid K-Cl cotransporter 2
AU167923	NP_036433 atrophin-1 interacting protein 1; likely ortholog of mouse activin
AU168600	NP_001356 postsynaptic density protein 95; discs large homolog 4
AU169110	NP_653229 chromosome 20 open reading frame 140
AU169163	NP_079455 plexin A2; plexin 2; plexin-A2
AU169168	NP_000654 4-aminobutyrate aminotransferase precursor; GABA transferase
AU169236	NP_055964 ral guanine nucleotide dissociation stimulator-like 1; RalGDS-like
AU169784	NP_006651 ClpX caseinolytic protease X homolog
AU170423	NP_004938 dedicator of cytokinesis 3; dedicator of cyto-kinesis 3
AU170444	NP_060111 kinesin family member 21A; NY-REN-62 antigen
AU170950	NP_055736 latrophilin 1; lectomedin-2
AU171067	NP_002367 MAP/microtubule affinity-regulating kinase 3
AU171249	NP_002232 potassium inwardly-rectifying channel J10; inward rectifier K+
AU171332	NP_008954 Rap guanine nucleotide exchange factor (GEF) 4; exchange protein
AU171374	XP_166420 PREDICTED: phosphatase and actin regulator 1
AU171447	NP_004647 BRCA1 associated protein-1; ubiquitin carboxy-terminal hydrolase
AU171481	NP_003673 MAP-kinase activating death domain-containing protein isoform d
AU176665	NP_006464 TAF6-like RNA polymerase II; p300/CBP-associated factor
AU177030	NP_004662 protein inhibitor of activated STAT X isoform beta
AU177167	NP_079170 hypothetical protein FLJ14075
AU177176	NP_055566 metastasis suppressor 1; missing in metastasis
AU177261	NP_006141 LIM domain only 6
AU177627	NP_009055 utrophin; dystrophin-related protein
AU177751	NP_443102 sorting nexin associated golgi protein 1; sorting nexin 18
AU180234	NP_775105 SNF2 histone linker PHD RING helicase; 2610103K11Rik
BJ004132	NP_004514 kinesin family member 11; thyroid receptor interacting protein 5
BJ007726	NP_689742 G protein-coupled receptor 155
BJ009104	NP_005515 hairy and enhancer of split 1; transcription factor HES-1
BJ000038	NP_002530 ornithine decarboxylase 1
BJ002908	NP_663788 Rho guanine nucleotide exchange factor 7 isoform b; SH3
BJ002997	XP_291015 PREDICTED: likely homolog of rat kinase D-interacting substance
BJ003764	NP_003609 mitogen-activated protein kinase kinase kinase kinase 3; germinal
BJ008665	NP_008936 transducin-like enhancer protein 4; enhancer of split groucho 4
BJ008783	NP_031401 TAR DNA binding protein; TAR DNA-binding protein-43
BJ008810	NP_000457 peroxisome biogenesis factor 1
BJ008817	NP_009160 protein kinase C and casein kinase substrate in neurons 2; pacsin 2
BJ010925	NP_055833 KIAA1117 protein
BJ011691	NP_004332 carbamoylphosphate synthetase 2/aspartate
BJ012243	NP_073585 tensin-like SH2 domain containing 1; tensin-like SH2
BJ012254	NP_001232 cyclin T2 isoform a; cyclin T2a; cyclin T2b; SDS-stable
BJ012312	NP_004929 death-associated protein kinase 1
BJ004544	NP_116045 DEAH (Asp-Glu-Ala-His) box polypeptide 37; DEAD/DEAH box helicase
BJ000375	NP_004422 ephrin receptor EphA2; epithelial cell receptor protein tyrosine
BJ001056	NP_653169 chromosome 14 open reading frame 9
BJ004731	NP_055662 NICE-4 protein
BJ005366	NP_079410 hypothetical protein FLJ12178
BJ009760	NP_002304 actin-binding LIM protein 1 isoform a; actin-binding LIM protein
BJ009771	XP_048747 PREDICTED: KIAA1223 protein
BJ011904	NP_079047 hypothetical protein FLJ13848
BJ012648	NP_065799 KIAA1287 protein
BJ013377	NP_115612 ubiquitin specific protease 48; ubiquitin specific protease 31
BJ014248	NP_004835 SH3-domain binding protein 5 (BTK-associated); SH3 binding protein
BJ002146	NP_003122 serum response factor (c-fos serum response element-binding
BJ005545	NP_055389 nuclear autoantigen; cell cycle S/G2 nuclear autoantigen
BJ005642	NP_055630 thyroid hormone receptor-associated protein; thyroid hormone
BJ005657	NP_079350 Fraser syndrome 1 isoform 1; extracellular matrix protein
BJ005724	NP_036412 FtsJ homolog 1 isoform a; rRNA (uridine-2'-O-)-methyltransferase
BJ006410	NP_061908 protocadherin 18 precursor; protocadherin 68-like protein
BJ007259	NP_037528 ubiquitin specific protease 25; ubiquitin specific protease USP25
BJ010058	NP_542768 intermediate filament-like protein MGC:2625 isoform 2; intermediate
BJ013886	NP_061031 ubiquitin specific protease 49
BJ000816	NP_005582 meiotic recombination 11 homolog A isoform 1; double-strand break
BJ000842	XP_034594 PREDICTED: KIAA1604 protein
BJ007320	NP_009135 DEAD (Asp-Glu-Ala-Asp) box polypeptide 20; DEAD-box protein DP103
BJ007326	NP_003172 transcription factor T; T brachyury-like; T brachyury (mouse)
BJ008255	XP_375553 PREDICTED: KIAA0963
BJ008256	XP_371277 PREDICTED: similar to RIKEN cDNA C230093N12
BJ011040	NP_005325 host cell factor C1 (VP16-accessory protein)
BJ013919	NP_037452 host cell factor C2; host cell factor 2
BJ013939	NP_002870 RAD52 homolog isoform alpha; recombination protein RAD52
BJ011306	NP_002664 plexin B1; plexin 5; semaphorin receptor
BJ020308	NP_031375 PAX transcription activation domain interacting protein 1 like
BJ014506	NP_115635 hypothetical protein DKFZp434F054
BJ021405	NP_714941 nucleoporin 62kDa; nuclear pore glycoprotein p62
BJ017567	XP_033173 PREDICTED: protocadherin 19
BJ025664	NP_054868 HSPC049 protein
BJ025751	NP_787072 exocyst complex 84-kDa subunit
AU240484	NP_000129 fibrillin 1
AU242782	NP_695003 hypothetical protein MGC32065
AU244152	NP_055986 exportin 6; RAN binding protein 20
AJ457222	NP_003277 DNA topoisomerase I; type I DNA topoisomerase
AJ457493	NP_055383 UDP-N-acetyl-alpha-D-galactosamine:polypeptide
BJ493563	NP_699202 PDZ domain containing ring finger 1
BJ493781	NP_004973 potassium inwardly-rectifying channel J8; inwardly rectifying
BJ495426	NP_001388 endothelin converting enzyme 1
BJ496901	NP_055784 KIAA0893 protein
BJ497591	NP_000471 adenosine monophosphate deaminase (isoform E)
BJ499461	NP_000427 3-oxoacid CoA transferase 1 precursor; succinyl-CoA:3-ketoacid-CoA
BJ500895	NP_056986 progestin-induced protein; ubiquitin-protein ligase; hyperplastic
BJ501370	NP_055966 DIP2-like protein isoform a; disco-interacting protein 2 (Drosophila)
BJ501585	NP_064583 GK001 protein
BJ502508	XP_097736 PREDICTED: chromosome 20 open reading frame 82

S9: List of asymmetric divergent genes of *Oryzias latipes* with significantly lower distance to the human ortholog than other fish species.

BJ503567	NP_062826 methyltransferase like 3; putative methyltransferase; 2310024F18Rik
BJ506101	XP_499556 PREDICTED: hypothetical protein FLJ10747
BJ507473	NP_958839 growth arrest-specific 7 isoform c
BJ487313	NP_003301 tumor suppressing subtransferable candidate 1
BJ488812	NP_114141 hemicentin; fibulin 6
BJ489466	NP_001805 cathepsin C isoform a preproprotein; dipeptidyl-peptidase I
BJ489868	NP_003588 TGFB inducible early growth response 2
BJ489926	NP_005112 thyroid hormone receptor associated protein 1
BJ490421	NP_054722 DEAH (Asp-Glu-Ala-His) box polypeptide 38; pre-mRNA splicing factor
BJ490861	NP_004289 nucleoporin 155kDa isoform 2; nuclear pore complex protein Nup155
BJ490986	XP_039796 PREDICTED: TRAF2 and NCK interacting kinase
BJ512308	NP_945341 N-acyl-phosphatidylethanolamine-hydrolyzing phospholipase D
BJ516424	NP_001026 ryanodine receptor 2
BJ517069	NP_009169 peroxisomal membrane protein 4 isoform a; 24 kDa peroxisomal
BJ517858	NP_005917 microfibrillar-associated protein 1
BJ517869	NP_060857 spermatid perinuclear RNA-binding protein
BJ518797	NP_061335 kelch-like 9
BJ519069	NP_004244 phospholipase A2-activating protein
BJ519269	NP_004321 BCL2/adenovirus E1B 19kD interacting protein 2; BCL2/adenovirus E1B
BJ519895	NP_009060 zinc finger protein of the cerebellum 2; Zic family member 2
BJ520045	NP_065901 Ran-binding protein 10
BJ520537	NP_000181 hydroxymethylbilane synthase; porphobilinogen deaminase
BJ522173	NP_001370 DNA (cytosine-5-)-methyltransferase 1; DNA methyltransferase 1
BJ524171	NP_056201 component of oligomeric golgi complex 4; complexed with Dor1p;
BJ524506	NP_000170 mutS homolog 6; G/T mismatch-binding protein; mutS (E. coli) homolog
BJ525224	NP_060895 phosphatidylinositol 4-kinase type II
BJ525915	NP_722523 sorting nexin 14 isoform a
BJ526240	NP_002379 minichromosome maintenance protein 3; cervical cancer
BJ527420	NP_003571 neutral sphingomyelinase (N-SMase) activation associated factor
BJ527642	NP_065943 deltex 2; deltex (Drosophila) homolog 2
BJ527988	NP_064505 UDP-glucose ceramide glucosyltransferase-like 1
BJ530427	NP_065165 putative homeodomain transcription factor 2
BJ537125	NP_056016 block of proliferation 1
BJ539899	NP_008993 nuclear phosphoprotein similar to S. cerevisiae PWP1
BJ540847	NP_064708 hypothetical protein DKFZp434K046
BJ542973	NP_005137 squamous cell carcinoma antigen recognized by T cells 1; U4/U6.U5
BJ543391	NP_000981 ribosomal protein L27a; 60S ribosomal protein L27a
AB095501	NP_055748 SEC31-like 1 isoform 1; yeast Sec31p homolog; protein-transport
BJ704133	NP_055478 jumonji domain containing 2A
BJ704447	NP_005737 pre-B-cell colony enhancing factor 1 isoform a
BJ704659	NP_056323 TCDD-inducible poly(ADP-ribose) polymerase
BJ704694	NP_060631 NAD synthetase 1; glutamine-dependent NAD synthetase
BJ705222	NP_002949 RYK receptor-like tyrosine kinase precursor; hydroxyaryl-protein
BJ705340	NP_001109 type I adenylate cyclase activating polypeptide receptor precursor
BJ706016	NP_060635 polybromo 1
BJ706074	NP_002145 heat shock 70kDa protein 4 isoform a; heat shock 70kD protein 4
BJ706077	NP_006416 step II splicing factor SLU7
BJ706164	NP_061936 echinoderm microtubule associated protein like 4
BJ706497	NP_055524 ubiquitin specific protease 34
BJ706791	XP_027330 PREDICTED: RNA binding motif protein 25
BJ707051	NP_061832 DAZ associated protein 1 isoform b; deleted in azoospermia
BJ707934	NP_057569 chromosome 6 open reading frame 55; My012 protein
BJ707948	NP_004032 arrestin beta 1 isoform A
BJ707979	NP_056988 translation initiation factor IF2
BJ708027	NP_003165 supervillin isoform 1; membrane-associated F-actin binding protein
BJ708068	NP_073738 sperm protein SSP411; transcript increased in spermiogenesis 78
BJ708573	NP_003899 eukaryotic translation initiation factor 2 beta; eukaryotic
BJ708635	NP_002960 mitogen-activated protein kinase 12; p38gamma; stress-activated
BJ709021	NP_110417 T-cell immunomodulatory protein
BJ709423	NP_620148 ectonucleoside triphosphate diphosphohydrolase 8; apyrase
BJ709607	NP_001922 dihydrolipoamide S-acetyltransferase (E2 component of pyruvate
BJ709687	XP_379983 PREDICTED: similar to KIAA0010
BJ709692	NP_006151 neurogenic differentiation 2
BJ710203	XP_376680 PREDICTED: KIAA1718 protein
BJ711169	NP_149989 hypothetical protein LOC92922
BJ711261	NP_000541 tyrosinase-related protein 1
BJ711276	NP_003070 SWI/SNF-related matrix-associated actin-dependent regulator
BJ711340	NP_036461 monocyte to macrophage differentiation-associated
BJ711358	NP_009204 repressor of estrogen receptor activity; B-cell associated protein
BJ712018	NP_057191 zinc finger RNA binding protein; M-phase phosphoprotein homolog
BJ712122	NP_542131 suppressor of actin mutations 2-like isoform a; dJ1033B10.5; SAC2
BJ712436	NP_075067 vacuolar protein sorting 33A
BJ712542	NP_002491 neurogenic differentiation 1; Neurogenic differentiation
BJ712805	NP_009180 Kruppel-like factor 12 isoform a; KLF12 zinc finger transcriptional
BJ713400	NP_006480 neuro-oncological ventral antigen 1 isoform 2; paraneoplastic Ri
BJ713568	NP_064507 potassium channel modulatory factor 1; differentially expressed in
BJ714237	NP_002582 cytosolic phosphoenolpyruvate carboxykinase 1; phosphoenolpyruvate
BJ714382	NP_071903 limb region 1 protein; limb region 1
BJ715164	NP_002878 arginyl-tRNA synthetase
BJ715687	NP_057300 debranching enzyme homolog 1; debranching enzyme (S. Cerevisiae)
BJ717464	NP_003240 thimet oligopeptidase 1
BJ719118	NP_658985 apolipoprotein A-I binding protein; apoA-I binding protein
BJ719534	NP_001121 amino-terminal enhancer of split isoform b
BJ720039	XP_045423 PREDICTED: KIAA0701 protein
BJ720872	NP_056380 zinc finger protein 294
BJ721289	NP_001816 sarcomeric mitochondrial creatine kinase precursor; basic-type
BJ723358	NP_006074 RED protein; RD element; prer protein; IK factor;
BJ724303	NP_001924 dihydrolipoamide S-succinyltransferase (E2 component)
BJ724521	NP_115497 implantation-associated protein
BJ726625	NP_077022 mitogen-activated protein kinase associated protein 1; ras
BJ727296	NP_694856 ankyrin repeat domain protein 15; kidney ankyrin repeat-containing
BJ727744	NP_115607 hypothetical protein FLJ22875
BJ728080	NP_733751 myeloid/lymphoid or mixed-lineage leukemia 3
BJ728082	NP_001264 chromodomain helicase DNA binding protein 4; Mi-2b
BJ728141	NP_055262 ADP-ribosylation factor interacting protein 1 (arfaptin 1)
BJ726961	NP_057128 CGI-105 protein
BJ728281	NP_055615 engulfment and cell motility 1 isoform 1; ced-12 homolog 1
BJ728299	NP_003211 transcription factor AP-2 alpha; activating enhancer-binding
BJ728451	NP_004586 spermine synthase; spermidine aminopropyltransferase
BJ728665	NP_055596 Rb1-inducible coiled coil protein 1

S9: List of asymmetric divergent genes of *Oryzias latipes* with significantly lower distance to the human ortholog than other fish species.

BJ729021	NP_001096 activin A type I receptor precursor; hydroxyalkyl-protein kinase
BJ729302	NP_004836 CTP:phosphocholine cytidylyltransferase b
BJ729374	NP_000993 ribosomal protein P0; 60S acidic ribosomal protein P0
BJ729829	NP_056203 chromosome 6 open reading frame 109; natural killer cell-specific
BJ729946	NP_003015 intersectin 1 isoform ITSN-l; SH3 domain protein-1A
BJ729976	NP_002309 lysyl oxidase-like 2
BJ730502	NP_115604 male sterility domain containing 2
BJ730543	NP_620309 B-cell lymphoma 6 protein; B-cell CLL/lymphoma-6
BJ730728	NP_056416 hect domain and RLD 4
BJ730756	NP_005603 RE1-silencing transcription factor; repressor binding to the X2
BJ731205	NP_006631 MLL septin-like fusion; septin D1; MLL septin-like fusion
BJ731438	NP_006345 transcriptional adaptor 3-like isoform a
BJ732302	NP_004521 matrix metalloproteinase 2 preproprotein; gelatinase neutrophil
BJ732615	NP_004963 Janus kinase 2; tyrosine-protein kinase JAK2
BJ732869	NP_066025 CEGP1 protein
BJ732904	XP_060020 PREDICTED: hypothetical protein BC016683
BJ733043	NP_003244 T-cell lymphoma invasion and metastasis 1; human T-lymphoma invasion
BJ733298	NP_036585 transducin (beta)-like 2 isoform 1; Williams-Beuren syndrome
BJ733387	NP_000611 nitric oxide synthase 1 (neuronal)
BJ733416	NP_149105 MADP-1 protein; U11/U12 snRNP 31K
BJ733490	NP_006256 RAD21 homolog; protein involved in DNA double-strand break repair
BJ733549	NP_055336 SH3-domain binding protein 4
BJ734294	NP_009133 A-kinase anchor protein 10 precursor; dual-specificity A-kinase
BJ734614	NP_004555 PET112-like; PET112 (yeast homolog)-like
BJ734780	NP_005923 mitochondrial intermediate peptidase
BJ734934	NP_055535 serine/threonine kinase 2; Ste20-like kinase; SNF1 sucrose
BJ735209	NP_006627 methylene tetrahydrofolate dehydrogenase 2 precursor; NAD-dependent
BJ735211	NP_056442 LDL receptor adaptor protein
BJ735266	NP_001027 ryanodine receptor 3
BJ735363	NP_057422 Ran binding protein 11
BJ735367	NP_057515 mitogen-activated protein kinase 8 interacting protein 2 isoform 2
BJ735433	NP_002102 huntingtin
BJ735889	NP_079094 ubiquitin-activating enzyme E1-domain containing 1 isoform 1
BJ736362	NP_003403 zinc finger protein of the cerebellum 1; Zic family member 1
BJ736836	NP_005511 heterogeneous nuclear ribonucleoprotein H1
BJ736871	NP_056051 latrophilin 3; latrophilin homolog 3 (cow); lectomedin 3
BJ736888	NP_005095 bromodomain containing protein 2; female sterile homeotic-related
BJ737008	NP_064555 nicalin; nicastrin-like protein
BJ737046	NP_001339 death-associated protein kinase 3
BJ737071	NP_116232 fibrinogen C domain containing 1
BJ737305	NP_002871 v-raf-1 murine leukemia viral oncogene homolog 1
BJ737548	NP_036322 formyltetrahydrofolate dehydrogenase isoform a
BJ737706	NP_689559 hypothetical protein MGC34680
BJ737712	NP_005299 G protein-coupled receptor kinase 5
BJ738539	NP_005105 heparan sulfate D-glucosaminyl 3-O-sulfotransferase 1 precursor
BJ738782	NP_057165 palladin; CGI-151 protein
BJ739066	NP_061921 HCV NS3-transactivated protein 1
BJ740453	NP_000897 natriuretic peptide receptor A/guanylate cyclase A
BJ741424	NP_112506 chromosome 20 open reading frame 18 isoform 2
BJ742023	NP_005324 holocytochrome c synthase (cytochrome c heme-lyase)
BJ742359	NP_000480 transcriptional regulator ATRX isoform 1; DNA dependent ATPase
BJ742416	NP_054727 JM1 protein
BJ743130	NP_002435 moesin
BJ746379	NP_116233 microtubule associated serine/threonine kinase-like
BJ748332	NP_003128 SFRS protein kinase 1; SR protein kinase 1
BJ748807	NP_659501 SH3 and cysteine rich domain 3
AV668349	NP_114402 N-myc downstream-regulated gene 3 isoform a; N-myc
AV668735	XP_047325 PREDICTED: Tho2
AV668786	XP_374996 PREDICTED: AMPK-related protein kinase 5
AV669002	NP_000515 5-hydroxytryptamine (serotonin) receptor 1A
AV669139	NP_055683 ring finger protein 10
AV670534	NP_149044 hippocampus abundant transcript 1; tetracycline transporter-like
AV670555	NP_005706 uronyl-2-sulfotransferase; uronyl 2-sulfotransferase
AV670991	NP_002219 v-jun avian sarcoma virus 17 oncogene homolog; Jun activation

S10: List of asymmetric divergent genes of *Oryzias latipes* with significantly higher distance to the human ortholog than other fish species.

GeneBank Acc#	annotation according to human (UniGene)
BJ518323	NP_055055 LIM domains containing 1
AU172100	NP_003861 IQ motif containing GTPase activating protein 1; rasGAP-like with IQ
BJ730618	NP_009009 LIM domain binding 3; Z-band alternatively spliced PDZ-motif
BJ493186	NP_001101 a disintegrin and metalloprotease domain 10
BJ728956	NP_000035 androgen receptor; dihydrotestosterone receptor
BJ000112	NP_003864 neuropilin 1
BJ713413	NP_000689 arachidonate 5-lipoxygenase
AU169472	NP_055078 YME1-like 1 isoform 3; ATP-dependent metalloprotease FtsH1 homolog
AJ410705	NP_005609 delta-like 1; delta-like 1 protein; delta (Drosophila)-like 1
AU168298	NP_065170 similar to aspartate beta hydroxylase (ASPH)
BJ705617	NP_001296 claudin 4; Clostridium perfringens enterotoxin receptor 1
BJ730796	NP_116231 hypothetical protein FLJ14803
AU170247	NP_055862 KIAA0090 protein
BJ714458	NP_055295 zinc finger protein 544
BJ738268	NP_055109 translocating chain-associating membrane protein
BJ705015	NP_006722 fukutin
BJ524561	NP_036440 F-box and leucine-rich repeat protein 11; F-box protein FBL11
AU168617	XP_106386 PREDICTED: KIAA1345 protein
BJ517379	NP_000254 alpha-N-acetylglucosaminidase
BJ008666	NP_057345 delta-tubulin
AU169692	XP_497656 PREDICTED: KIAA0962 protein
BJ728505	XP_371078 PREDICTED: similar to Galectin-3 binding protein precursor
BJ498785	NP_066192 claudin 9
BJ025006	NP_776163 hypothetical protein MGC50559
BJ502548	NP_002072 glypican 1 precursor
BJ513172	XP_292184 PREDICTED: similar to immune-responsive gene 1
